# Telehealth-Supported Exercise or Physical Activity Programs for Knee Osteoarthritis: Systematic Review and Meta-Analysis

**DOI:** 10.2196/54876

**Published:** 2024-08-02

**Authors:** Xiao-Na Xiang, Ze-Zhang Wang, Jing Hu, Jiang-Yin Zhang, Ke Li, Qi-Xu Chen, Fa-Shu Xu, Yue-Wen Zhang, Hong-Chen He, Cheng-Qi He, Si-Yi Zhu

**Affiliations:** 1 Rehabilitation Medicine Center and Institute of Rehabilitation Medicine West China Hospital Sichuan University Chengdu China; 2 Key Laboratory of Rehabilitation Medicine in Sichuan Province West China Hospital Sichuan University Chengdu China; 3 School of Rehabilitation Sciences West China School of Medicine Sichuan University Chengdu China; 4 Center of Statistical Research School of Statistics Southwestern University of Finance and Economics Chengdu China; 5 Joint Lab of Data Science and Business Intelligence School of Statistics Southwestern University of Finance and Economics Chengdu China; 6 West China Biomedical Big Data Center West China Hospital Sichuan University Chengdu China

**Keywords:** telehealth, knee osteoarthritis, physical activities, quality of life, systematic review and meta-analysis, systematic review, meta-analysis, knee, physical activity, exercise, chronic disease, chronic disease management, effectiveness, physical function, self-efficacy

## Abstract

**Background:**

The integration of telehealth-supported programs in chronic disease management has become increasingly common. However, its effectiveness for individuals with knee osteoarthritis (KOA) remains unclear.

**Objective:**

This study aimed to assess the effectiveness of telehealth-supported exercise or physical activity programs for individuals with KOA.

**Methods:**

A comprehensive literature search encompassing Embase, MEDLINE, CENTRAL, Web of Science, PubMed, Scopus, PEDro, GreyNet, and medRxiv from inception to September 2023 was conducted to identify randomized controlled trials comparing telehealth-supported exercise or physical activity programs to a control condition for KOA. Data were extracted and qualitatively synthesized across eligible studies, and a meta-analysis was performed to evaluate the effects. The study was reported according to PRISMA (Preferred Reporting Items for Systematic Reviews and Meta-Analyses) 2020.

**Results:**

In total, 23 studies met eligibility criteria, with 20 included in the meta-analysis. Results showed that telehealth-supported exercise or physical activity programs reduced pain (*g*=–0.39; 95% CI –0.67 to –0.11; *P*<.001), improved physical activity (*g*=0.13; 95% CI 0.03-0.23; *P*=.01), and enhanced physical function (*g*=–0.51; 95% CI –0.98 to –0.05; *P*=.03). Moreover, significant improvements in quality of life (*g*=0.25; 95% CI 0.14-0.36; *P*<.001), self-efficacy for pain (*g*=0.72; 95% CI 0.53-0.91; *P*<.001), and global improvement (odds ratio 2.69, 95% CI 1.41-5.15; *P*<.001) were observed. However, self-efficacy for physical function (*g*=0.14; 95% CI –0.26 to 0.53; *P*=.50) showed insignificant improvements. Subgroup analyses based on the World Health Organization classification of digital health (pain: *χ*^2^_2_=6.5; *P*=.04 and physical function: *χ*^2^_2_=6.4; *P*=.04), the type of teletechnology in the intervention group (pain: *χ*^2^_4_=4.8; *P*=.31 and function: *χ*^2^_4_=13.0; *P*=.01), and active or inactive controls (pain: *χ*^2^_1_=5.3; *P*=.02 and physical function: *χ*^2^_1_=3.4; *P*=.07) showed significant subgroup differences.

**Conclusions:**

Telehealth-supported exercise or physical activity programs might reduce knee pain and improve physical activity, physical function, quality of life, self-efficacy, and global improvement in individuals with KOA. Future research should consider longer implementation durations and assess the feasibility of incorporating wearables and standardized components into large-scale interventions to evaluate the effects.

**Trial Registration:**

PROSPERO CRD42022359658; https://www.crd.york.ac.uk/prospero/display_record.php?RecordID=359658

## Introduction

Osteoarthritis is a prevalent degenerative joint disease affecting more than 500 million individuals globally [1], with over 260 million individuals experiencing knee osteoarthritis (KOA) alone, resulting in significant health and socioeconomic burdens [2]. The prevalence of KOA among individuals aged 60 years is 26.8% [3], and projections indicate that by the year 2100, KOA will affect approximately 2.37 billion people aged 65 years and older and 866 million individuals aged 80 years and older worldwide [4]. At the early stage of KOA, pain and stiffness are the predominant symptoms; thus, management strategies aim to alleviate pain and increase functional capacities [5]. Physical activity reflects individuals’ overall activity levels, while exercise denotes a planned, structured, and repetitive subset of physical activity [6]. The level of daily physical activity correlates positively with the physical function and quality of life of patients with KOA while also controlling pain intensity [7]. Clinical guidelines advocate for physical activity and exercise as the first-line management strategy for KOA [2,8,9]. Exercise and physical activity interventions have been shown to alleviate KOA symptoms and delay functional impairment and eventual joint replacement [10,11]. However, gaps exist in the clinical application of active lifestyle and exercise for KOA, with a tendency to overly rely on medication and surgery [12]. Effective interventions are desperately needed to address decreased function associated with an inactive lifestyle and aging. However, in-person health care services, particularly physical therapy, can be expensive in terms of time and other costs, such as consultation fees and transportation, for individuals with incurable KOA requiring long-term intervention [13]. Furthermore, lacking motivation to maintain an active lifestyle is also a barrier since adherence to home-based exercise decreased by 94.7% at 3 months following discharge [14].

Telehealth is defined as “the delivery and facilitation of health and health-related services including medical care, provider and patient education, health information services, and self-care via telecommunications and digital communication technologies” [15,16]. An accumulating body of evidence suggests that telehealth-supported exercise interventions have been proven as a preferable form of intervention, especially due to the “social distancing” requirement imposed by the COVID-19 pandemic. Hence, the need for advice or interventions via telehealth has soared [17,18]. However, because of its complicated operating system, ambiguous instructions, and need for Wi-Fi or cellular data, digital rehabilitation may not be as beneficial as face-to-face rehabilitation for people with KOA who are typically older.

The modes of remote rehabilitation are diverse, and many design factors, such as reminders, supervision, and communication, are closely related to the ultimate intervention outcomes. The high heterogeneity in the design of existing clinical trials on remote rehabilitation poses challenges in comparing the effectiveness of methods and summarizing experiences. Several reviews have attempted to evaluate the efficacy of telehealth-supported exercise programs in individuals with KOA [19-21], but few have focused on the efficacy of telehealth-supported physical activity programs. Our previous meta-analysis (n=4) [21] indicated that internet-based rehabilitation relieved pain in patients with KOA, but its effect on physical function was unclear due to the limited inclusion of original studies. Regarding the treatment effect of computer- or virtual reality–supported exercise on patients with KOA, another meta-analysis (n=12) [22] found no improvement in physical function, which might result from heterogeneity in exercise programs and experimental design. Additionally, these reviews did not examine how telehealth-supported exercise programs affect physical activity, self-efficacy in coping with symptoms, or global improvement experienced by patients.

To enhance our comprehension of the impact of telehealth-supported exercise or physical activity programs in individuals with KOA, as examined by multiple studies [23-32], we conducted a systematic review and meta-analysis. The objective of this investigation was to assess the effect of telehealth-supported exercise or physical activity programs on pain, physical activity, physical function, self-efficacy, quality of life, and global improvement with a comprehensive bias assessment. Additionally, the study applied the World Health Organization (WHO) classification of digital health [33] to articulate the functionalities of each program and provided an analysis of the minimally important differences (MIDs), which are important considerations in clinical decision-making.

## Methods

### Selection Process, Search Strategy, and Eligibility Criteria

The review protocol was registered with PROSPERO (CRD42022359658) and reported according to the PRISMA (Preferred Reporting Items for Systematic Reviews and Meta-Analyses) recommendations, version 2020 (PRISMA checklist is present in Multimedia Appendix 1) [34]. Multimedia Appendix 2 contained a list of modifications to the study protocol. For randomized controlled trials (RCTs) published in English-language peer-reviewed journals, the following databases were searched: Embase (via OVID platform), MEDLINE (via OVID platform), CENTRAL (via the Cochrane Library), Web of Science, PubMed, Scopus, and PEDro from inception to September 2023. The specialist registers GreyNet (GreyNet International) and medRxiv (Cold Spring Harbor Laboratory) were searched for gray literature. To identify possibly pertinent studies, the reference lists of studies included in the full-text screening process were manually searched. We developed a search strategy, and the full search strategy is listed in Multimedia Appendix 3. The entire search process was assisted by a librarian from Sichuan University.

A wide definition of a telehealth-supported structured exercise or physical activity programs was established, covering interventions delivery via telephone (voice calls), SMS text messages, mobile app (app-based), internet (web-based), and wearable device (electronics). Studies were included within the Participants, Intervention, Comparison, Outcome, and Study Design (PICOS) framework (Textbox 1). Studies were excluded if the research simultaneously addressed other forms of arthritis or included unclear statistical data. Studies identified from literature research were imported into Review Manager (version 5.4; Nordic Cochrane Centre, Cochrane Collaboration). Titles and abstracts were independently screened by 2 authors (XNX and ZZW) to identify studies for full-text screening. Any disagreements were resolved through discussion under the guidance of a third reviewer (SYZ). The process for full-text screening remained consistent.

**Inclusion criteria according to the Participants, Intervention, Comparison, Outcome, and Study Design framework.**

**Participants:**

- Participants regardless of age with a diagnosis of knee osteoarthritis

**Intervention:**

- Telehealth-supported structured exercise or physical activity programs delivered by telephone, SMS, mobile app, internet, and wearable device, or applications combined with wearable devices

**Comparison:**

- Telehealth-supported programs without exercise or physical activity, waiting list, or nontelemedicine interventions (ie, usual care, conventional exercise programs, and patient education)

**Outcomes:**

- Primary outcomes were pain, physical activity, and physical function and secondary outcomes were quality of life, self-efficacy for pain and function, and overall global improvement

**Study design:**

- Randomized controlled trial

### Data Extraction

Two independent authors (ZZW and JYZ) extracted data (author, year of publication, country, participants, intervention content and duration, forms of monitoring, forms of telehealth, delivery model, the WHO classification of digital health, and results) with a standardized data template. Specifically, the WHO classification of digital health [33] was used to systematically categorize the telehealth interventions and support the synthesis of research and evidence. Accordingly, studies were classified into (1) interventions for clients, (2) interventions for health care providers, and (3) interventions for both clients and health care providers. Disagreements between the 2 reviewers were resolved through consensus, and if necessary, by consultation with a third reviewer (SYZ). For each outcome of interest, means, SDs, and sample sizes were extracted for each comparison. If SDs were missing for continuous data, other statistics (ie, 95% CI; SEs; and *t*, *F*, or *P* values) were used for the calculation of SD via the calculator tool from Review Manager.

### Data Synthesis and Analysis

The effect sizes of each study were quantified using standardized mean differences (SMDs), which were computed by dividing the difference in means between the 2 groups by the pooled SD of the measurement [35]. The SMDs for each study were obtained from the changes in outcome measures prior to and after the intervention indicating the intervention’s influence on the outcome measures.

Data analysis was performed with Review Manager and R (version 4.2.1; R Foundation for Statistical Computing). Hedges *g* with a 95% CI was used to analyze continuous variables, irrespective of whether specific outcomes were identified. Heterogeneity was assessed with Cochrane *Q* statistic (significance level at *P*<.10) and quantified with *I*^2^ (substantial heterogeneity at *I*^2^>50%) [36,37]. The presumed variability across the included studies led to the application of the random-effects model. The results of fixed-effect model results were reported when heterogeneity was absent (τ^2^=0). Otherwise, the between-study differences were explained with the results of the random-effects model. Egger regression test, Begg rank correlation test, and funnel plot of the primary outcomes were used to assess the potential publication bias [38]. If the test for asymmetry was significant, the trim-and-fill method was used to address missing studies and estimate the pooled effect to adjust for possible bias. Influence analysis was used to identify outliers. Sensitivity analyses were performed on primary outcomes to confirm robustness, using the fixed-effect model and implementing the “leave-one-out” method [39], excluding outliers. Methodological assistance was provided by a researcher from the MAGIC China Center or Cochrane China Center at West China Hospital, Sichuan University.

### Meta-Analysis

A meta-regression test and subgroup analysis of the primary outcomes (pain, physical activity, and physical function) were conducted to identify factors contributing to heterogeneity. The meta-regression test considered variables that might influence the intervention effect and heterogeneity. Based on the regression results, selected factors were used for the subgroup analysis. Hedges *g* cut-off points of 0.20, 0.50, and 0.80, respectively, represented a small, moderate, and large effect. A *P* value <.05 was deemed statistically significant [35]. Hedges *g* and representative SDs (pooled from the intervention and control groups in trials using the scale) were used to calculate the MIDs for primary outcomes [40], which were then compared to the reported MIDs. The anchor-based estimates were applied when no MID was reported [41].

### Quality Assessment

The Cochrane Collaboration’s risk of bias tool, specifically Vision 2, was used to assess bias. We assessed biases in the following domains: randomization process, deviations from intended interventions, missing outcome data, measurement of the outcome, and selection of the reported result [42]. Every element was classified as low, some concerns, or high risk. Furthermore, the PEDro scale was used to assess the quality of included studies [43].

### Quality of Evidence Assessment

The GRADE (Grading of Recommendations Assessment, Development, and Evaluation) approach was applied to evaluate the certainty of evidence for each outcome. The overall certainty of evidence for each outcome was graded as high, moderate, low, or very low. Evidence was downgraded by 1 level for each serious problem identified in the domains of risk of bias, inconsistency (substantial heterogeneity: *I*^2^>50%), indirectness, imprecision (such as small sample size), and publication bias [44].

## Results

### Study Selection

**Figure 1:**
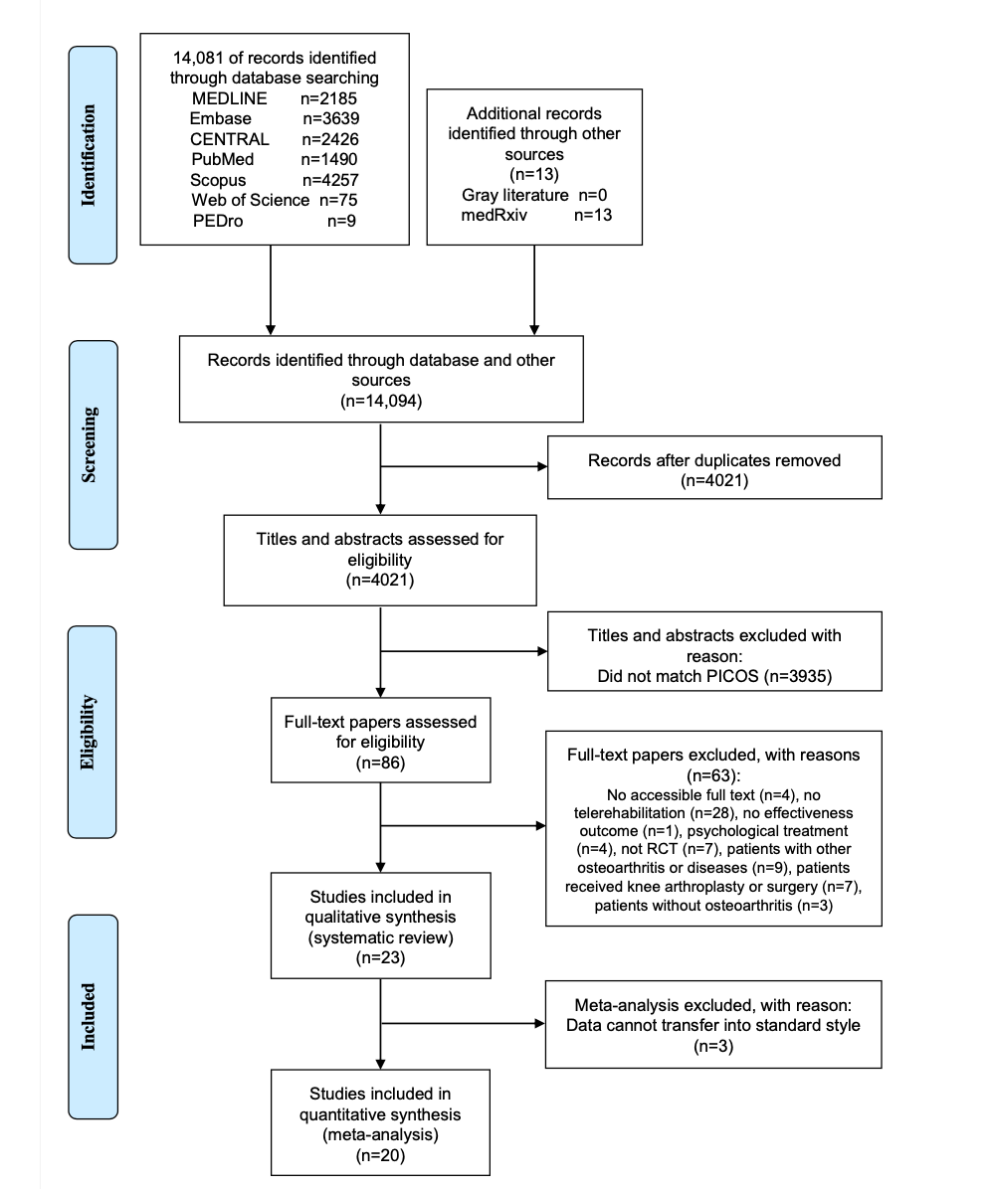
Flow diagram describing the literature review process. PICOS: Participants, Intervention, Comparison, Outcome, and Study Design; RCT: randomized controlled trial.

A total of 14,081 papers were initially identified from databases, with an additional 13 retrieved. After removing duplicates, 4021 records were screened for titles and abstracts. Subsequently, 86 full-text papers were assessed for eligibility. Of these, 23 papers [23-32,45-59] were included in the systematic review (Figure 1). Excluded studies at the full-text screening stage are listed in Multimedia Appendix 4, with reasons for exclusion. Three papers [46,58,59] were excluded from the meta-analysis due to uncalculated SDs, resulting in the inclusion of 20 (87%) papers.

### Characteristics of Included Studies

The characteristics of the included studies are presented in Multimedia Appendix 5 [24-32,45-54,56-59]. A total of 23 studies involving 3824 patients, of whom 2294 (60%) were female, were included in the systematic review. One study [26] exclusively examined the effect of telehealth-supported programs on the female population, while other studies recruited participants of both genders. Among these studies, 2 (9%) studies were conducted in Europe, 5 (22%) in North America, 5 (22%) in Asia, 9 (39%) in Australia, and 2 (9%) in Africa. The study period varied between 4 and 96 weeks, with 7 (30%) trials performing the telehealth-based intervention for less than 3 months, 6 (26%) for 3 to 6 months, and 10 (39%) for more than 6 months. A total of 13 (57%) studies provided telehealth-supported exercise programs, 4 (17%) provided physical activity programs, and 6 (26%) provided treatments in combination. Interventions were delivered through various digital technologies, including mobile app (n=4), telephone (n=4), internet-based platforms (n=4), SMS text messages (n=2), and combinations (n=9). Moreover, studies tested different factors related to program design and delivery. Various forms of monitoring were performed, such as reminder alone (n=4), remote coaching alone (n=3), remote monitoring alone (n=3), combined remote reminder and monitoring (n=2), combined remote coaching and monitoring (n=7), and fewer studies focused on combined remote reminder and coaching (n=1) or combined all (n=1). Concerning interaction during the delivery of telehealth, 8 (35%) studies delivered through virtual contact (non–face-to-face contact), no interacting contact occurred in 5 (22%) studies, 5 (22%) studies used mixed forms, and 1 (4%) study used in-person delivery.

The primary outcomes included pain, physical activity, and physical function. In the 23 included studies, pain was measured using the Western Ontario and McMaster Universities Osteoarthritis Index (WOMAC) pain subscale (n=12), Visual Analogue Scale (n=2), Knee Injury and Osteoarthritis Outcome Score (KOOS) pain subscale (n=5), or Numeric Pain Rating Scale (n=10). Physical activity levels were assessed using the Physical Activity Scale for the Elderly (n=6), time spent in daily moderate to vigorous physical activity (n=2), or the International Physical Exercise Questionnaire (n=1). Physical function was evaluated using the WOMAC function subscale (n=15), KOOS function subscale (n=2), Timed Up and Go test (n=6), or Ibadan Knee/Hip Osteoarthritis Outcome Measure (n=1).

The secondary outcomes included quality of life, self-efficacy for pain and function, and global improvement. Self-efficacy was measured using the Arthritis Self-Efficacy Scale, specifically its pain (n=6) and function (n=4) subscales. Quality of life was assessed using either the KOOS Quality of Life subscale (n=4) or the Assessment of Quality of Life (n=7). The global improvement was evaluated based on self-reported scores on a 7-point Likert scale (n=4). The outcome measurements identified in the included studies are detailed in Multimedia Appendix 5.

### Risk of Bias

The risk of bias analysis is presented in Multimedia Appendices 6 and 7 [24-32,45-54,56-59]. In total, 19 [24,25,29,30,32,45-59] of the 23 papers followed intention-to-treat analysis, while the remaining 4 [26-28,31] followed per-protocol analysis. Biases across 5 domains were identified and reported in corresponding studies. Concerns regarding the randomization process were noted in 6 (26%) studies [25,27,28,46,57,58]. Given the inherent difficulty in blinding participants in telehealth-based exercise and physical activity programs, concerns arose in the “deviations from intended interventions” domain among 12 (52.17%) studies [24,26-28,30,31,46,51,52,57-59]. All studies have reported strategies for incomplete outcome data. One (4%) study [26] exhibited a high risk of bias in outcome measurement due to insufficient information regarding blinded assessment. Concerns regarding the selection of the reported result were raised in 2 (9%) studies [49,51]. Overall, the risk of bias judgment indicated high risk in 2 (8.70%) studies [26,58] and low risks in 10 (43.48%) studies [29,32,45,47-50,53,54,56]. Meanwhile, the assessment outcomes of the PEDro scale are presented in Multimedia Appendix 7. Each of the 23 papers included in the study received PEDro scores exceeding 5 points. Notably, 12 papers obtained PEDro scores ranging between 6 and 8 points, thus falling within the classification of “good.” Furthermore, 11 papers achieved PEDro scores equal to or exceeding 9 points, indicating an “excellent” quality level.

### Main Analyses About Effects of Telehealth-Based Exercise or Physical Activity Programs

#### Overview

Meta-analysis results of the effects of telehealth-based exercise or physical activity programs on primary outcomes are presented in Table 1. The GRADE summary of findings is listed in Multimedia Appendix 8.

**Table 1:** Meta-analysis results of the primary effects of telehealth-based exercise or physical activity programs.

Primary effects	RCTs^a^, n	Hedges *g* (95% CI)	*P* value of Egger regression test	*P* value of Begg rank correlation test	Calculated MID^b^	Reported MID	Quality of evidence (GRADE^c^)^d^
Pain	19	–0.39 (–0.67 to –0.11)	.41	.92	1.3	2.0	⊕⊕⊖⊖^e,f^
Physical activity	9	0.13 (0.03 to 0.23)	.46	.40	9.0	46.0	⊕⊕⊖⊖^e,g^
Physical function	18	–0.51 (–0.98 to –0.05)	.19	.73	5.3	10.1	⊕⊕⊖⊖^e,h^

^a^RCT: randomized controlled trial.

^b^MID: minimally important difference.

^c^GRADE: Grading of Recommendations Assessment, Development and Evaluation.

^d^GRADE Working Group grades of evidence: High quality (⊕⊕⊕⊕): Further research is very unlikely to change our confidence in the estimate of effect. Moderate quality (⊕⊕⊕⊖): Further research is likely to have an important impact on our confidence in the estimate of effect and may change the estimate. Low quality (⊕⊕⊖⊖): Further research is very likely to have an important impact on our confidence in the estimate of effect and is likely to change the estimate. Very low quality (⊕⊖⊖⊖): We are very uncertain about the estimate.

^e^Downgraded for risk of bias: Participants and personnel were unblended.

^f^Downgraded for inconsistency: Considerable heterogeneity (*I*^2^=83%).

^g^Downgraded for imprecision: Small sample size [54].

^h^Downgraded for inconsistency: Considerable heterogeneity (*I*^2^=87%).

#### Pain

**Figure 2:**
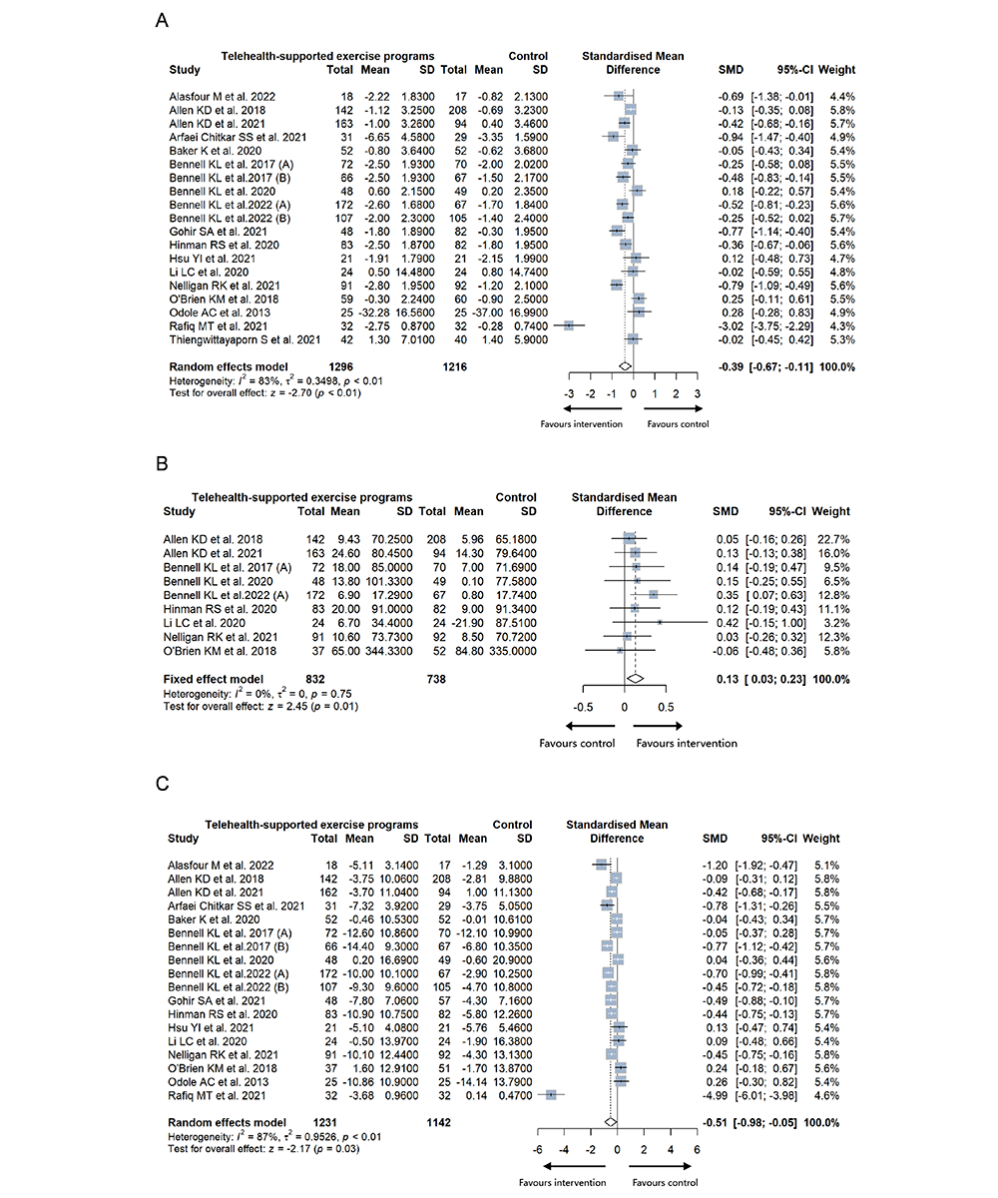
Forest plot of (A) pain, (B) physical activity, and (C) physical function.

In 19 studies, a significant difference and a small effect size were observed (n=2512; *g*=–0.39; 95% CI –0.67 to –0.11; *P*<.001; forest plot Figure 2A [24,26-32,45,47-54,56,57]), indicating a favorable impact of the telehealth-based intervention on pain. However, substantial heterogeneity was noted (*I*^2^=83%; τ^2^=0.3498; *P*<.001). The calculated MID of pain was 1.3, which was smaller than the reported MID (2.0 units for the WOMAC pain subscale) [60]. Overall, the evidence suggests a low certainty that telehealth-based exercise or physical activity programs lead to a small yet statistically significant reduction in pain, although the clinical significance of this reduction might be limited.

#### Physical Activity

The result of meta-analysis favored the telehealth-based intervention in promoting physical activity level of the population with KOA (n=1570; *g*=0.13; 95% CI 0.03-0.23; *P*=.01; forest plot Figure 2B) with negligible heterogeneity (*I*^2^=0%; τ^2^=0; *P*=.75). The calculated MID for physical activity, determined by Physical Activity Scale for the Elderly, was 9.0, which was less than the reported MID of 46.0 units [61]. Overall, the evidence was of low certainty and suggested that the telehealth-based programs might increase physical activity in a significant and very small way but not in a clinically meaningful manner.

#### Physical Function

The meta-analysis supported the telehealth-based intervention with a Hedges *g* effect size of –0.51 (n=2373; 95% CI –0.98 to –0.05; *P*=.03; forest plot Figure 2C) with considerable heterogeneity (*I*^2^=87%; τ^2^=0.9526; *P*<.001) for improving physical function. The reported MID (10.1 units for the WOMAC physical function subscale) was higher than the calculated MID of 5.3 [62]. Overall, there was low-certainty evidence to suggest that telehealth-based programs could improve the physical function of the population with KOA to a moderately significant degree, though not reaching a clinically meaningful.

#### Secondary Outcomes

Compared to populations in control groups, a larger improvement was observed in terms of secondary outcomes within the intervention groups. These included quality of life (n=1301; *g*=0.25; 95% CI 0.14-0.37; *P*<.001; heterogeneity: *I*^2^=5%; τ^2^=0.0033; *P*=.39; see “Quality of life” in Multimedia Appendix 9 [24,25,27,29,48-54]), self-efficacy for pain (n=1337; *g*=0.73; 95% CI 0.52-0.94; *P*<.001; heterogeneity: *I*^2^=4%; τ^2^=0.0056; *P*=.39; see “Self-efficacy for pain” in Multimedia Appendix 9), and global improvement (n=1042; odds ratio 2.69, 95% CI 1.41-5.15; *P*<.001; heterogeneity: *I*^2^=79%; τ^2^=0.3296; *P*<.001; see “Global improvement” in Multimedia Appendix 9). However, a nonsignificant trend and moderate heterogeneity were observed for self-efficacy for physical function (n=578; *g*=0.14; 95% CI –0.26 to 0.53; *P*=.50; heterogeneity: *I*^2^=52%; τ^2^=0.0833; *P*=.10; see “Self-efficacy for physical function” in Multimedia Appendix 9).

### Meta-Regression and Subgroup Analysis

A meta-regression test was conducted for several relative factors that might affect the intervention effect and heterogeneity, including coaching, monitoring, reminders, delivery form, intervention duration, sample size, quality of study, region of study, and other factors; details and results are presented in Multimedia Appendix 10. Notably, the regression results in type of teletechnology, WHO classification, and active or inactive control were significant.

**Figure 3:**
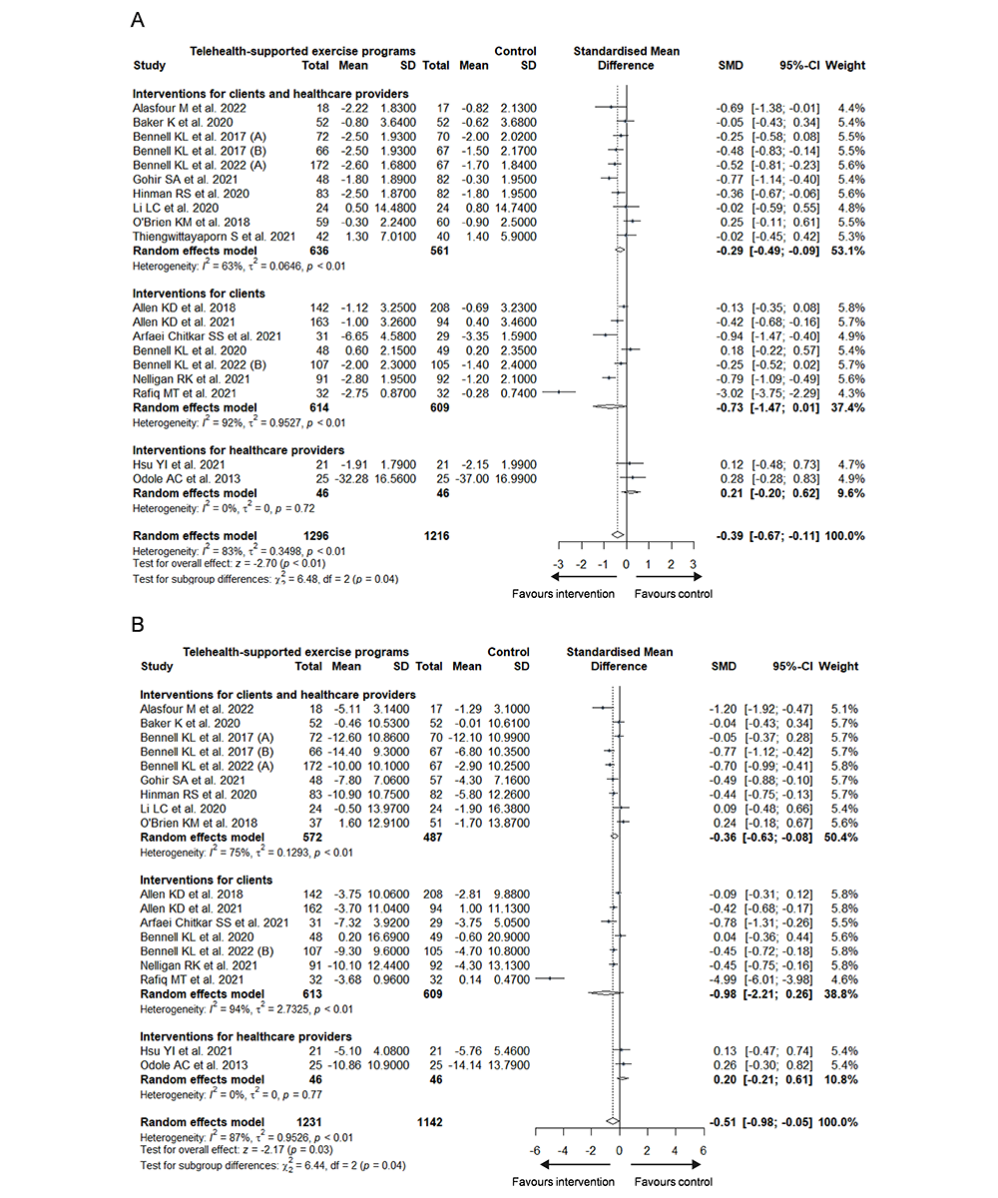
Subgroup analysis of (A) pain and (B) physical function based on the World Health Organization classification.

Subgroup analyses based on the WHO classification revealed significant differences in pain (*χ*^2^_2_=6.5; *P*=.04; Figure 3A [24-32,45,47-54,56,57]) and physical function (*χ*^2^_2_=6.4; *P*=.04; Figure 3B). Specifically, within the subgroup of interventions for clients and health care providers, telehealth-based intervention demonstrated significant effects on both pain (*g*=–0.29; 95% CI –0.49 to –0.09; heterogeneity: *I*^2^=63%; τ^2^=0.0646; *P*<.001) and physical function (*g*=–0.36; 95% CI –0.63 to –0.08; heterogeneity: *I*^2^=75%; τ^2^=0.1293; *P*<.001).

**Figure 4:**
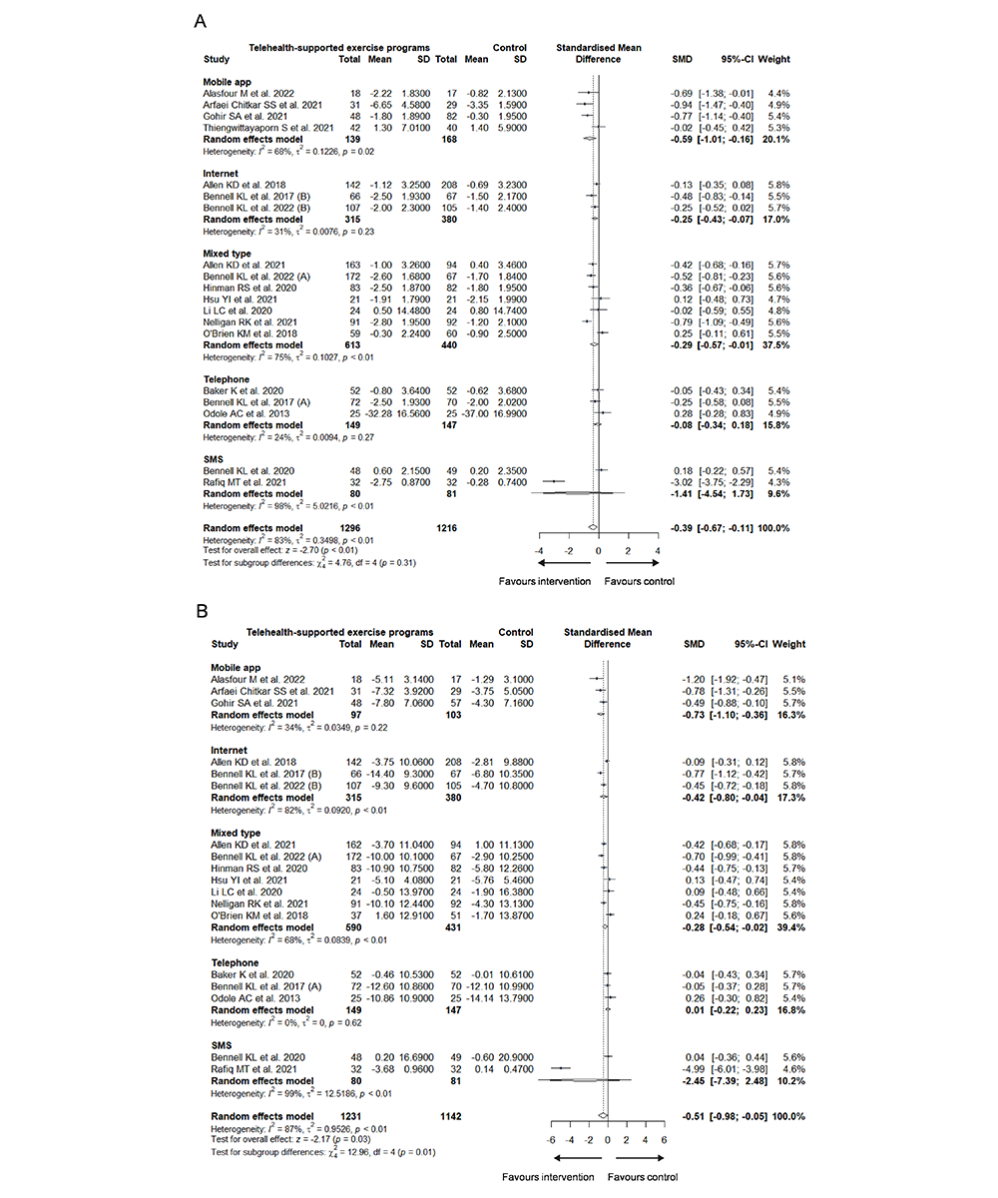
Subgroup analysis of (A) pain and (B) physical function based on the type of digital technology.

As for the type of digital technologies applied in the programs, the subgroup differences were significant in physical function (*χ*^2^_4_=13.0; *P*=.01; Figure 4B [24-32,45,47-54,56,57]) but not in pain (*χ*^2^_4_=4.8; *P*=.31; Figure 4A). Significant improvements were noticed in physical function across mobile app subgroup (*g*=–0.73; 95% CI –1.10 to –0.36; heterogeneity: *I*^2^=34%; τ^2^=0.0349; *P*=.22), internet subgroup (*g*=–0.42; 95% CI –0.80 to –0.04; heterogeneity: *I*^2^=82%; τ^2^=0.0920; *P*<.001), and mixed type of intervention (*g*=–0.28; 95% CI –0.54 to –0.02; heterogeneity: *I*^2^=68%; τ^2^=0.0839; *P*<.001).

**Figure 5:**
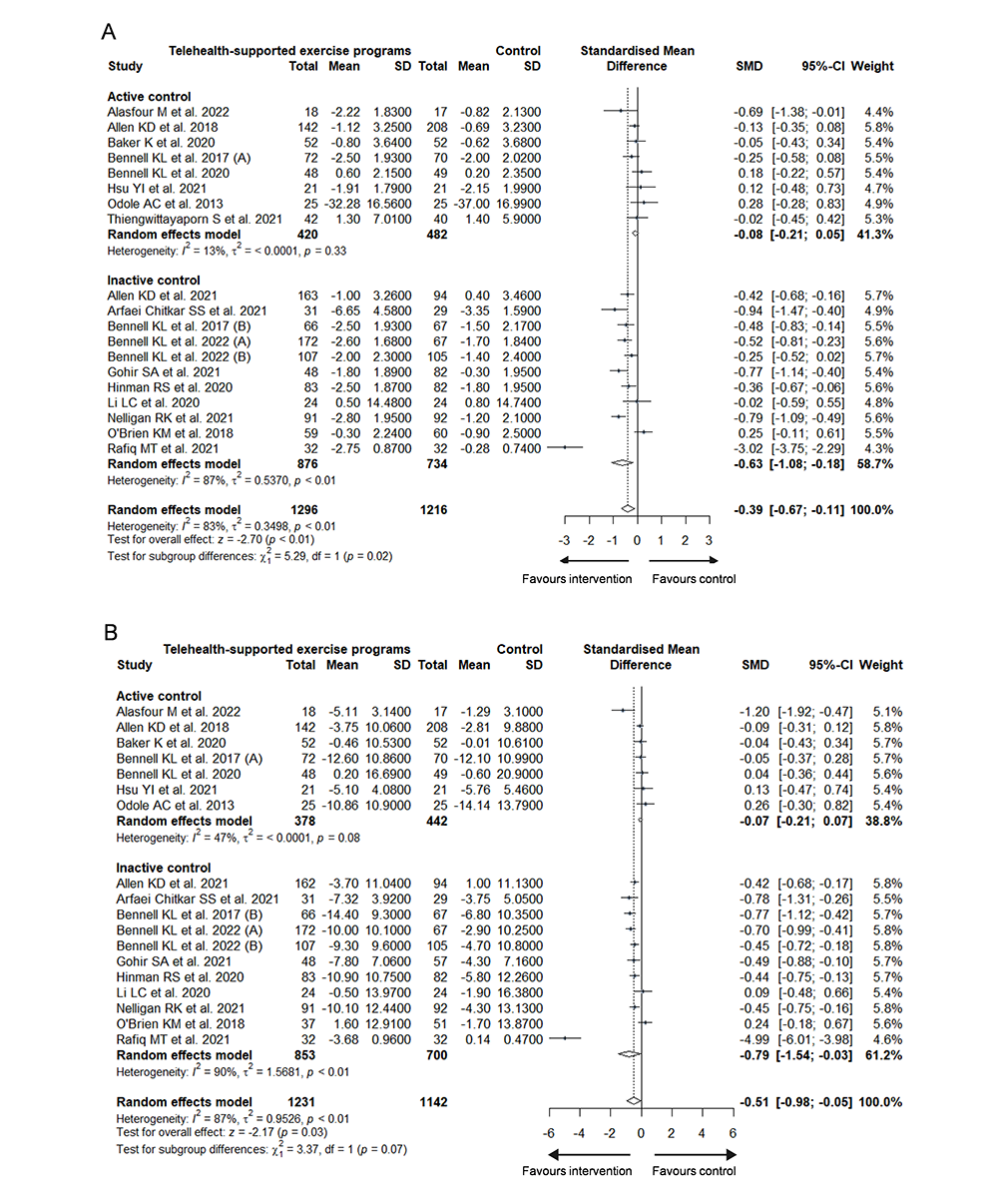
Subgroup analysis of (A) pain and (B) physical function based on the control group.

The forms of intervention in the control groups were divided into active controls (ie, exercise, physical therapy, pharmacotherapy, and self-management) or inactive controls (ie, education, usual care, and waitlist), which led to subgroup differences (pain: *χ*^2^_1_=5.3; *P*=.02; Figure 5A [24-32,45,47-54,56,57] and physical function: *χ*^2^_1_=3.4; *P*=.07; Figure 5B). Compared with inactive control groups, statistically significant pain reduction (*g*=–0.63; 95% CI –1.08 to –0.18; heterogeneity: *I*^2^=87%; τ^2^=0.5370; *P*<.001) and function improvement (*g*=–0.79; 95% CI –1.54 to –0.03; heterogeneity: *I*^2^=90%; τ^2^=0.9526; *P*<.001) were found in the intervention groups, while the differences between active controlled groups and intervention groups were not significant.

### Outliers and Influence Analysis

Multimedia Appendix 11 [24-32,45,47-54,56,57] demonstrates the Baujat plot and influence analyses. The study from Rafiq et al [28] in the pain category was identified as a possible outlier. Meanwhile, studies of Bennell et al [24], Rafiq et al [28], and Allen et al [45] were considered as outliers for physical function.

### Sensitivity Analysis

In the fixed-effect model result, a significant difference with a small effect size favoring telehealth-based intervention for pain relief was observed (see “Fix-effects model of pain” in Multimedia Appendix 12 [24,26-32,45,47-54,56,57]). After removing the outlier study [28], the pain relief effect size remained small (*g*=–0.28; 95% CI –0.44 to –0.11; see “Leave-one-out” analysis of pain” in Multimedia Appendix 12) with reduced heterogeneity (*I*^2^=69%; τ^2^=0.2849; *P*<.001). Furthermore, even after excluding studies with an overall high risk of bias and the outlier, the effect size for pain relief remained small (*g*=–0.29; 95% CI –0.47 to –0.11; see “Sensitivity analysis omitted studies with high risk of bias and outliers of pain” in Multimedia Appendix 12) and was presented with heterogeneity (*I*^2^=71%; τ^2^=0.0846; *P*<.001), indicating the robustness of the finding.

For physical function, the positive effect of the telehealth-based intervention was supported by the result of meta-analysis using the fixed-effect model (see “Fix-effects model of physical function” in Multimedia Appendix 12). Subsequent “leave-one-out” analyses confirmed the overall significant effect size for physical function improvement, which remained small (*g*=–0.30; 95% CI –0.47 to –0.13; see “Leave-one-out” analyses of physical function” in Multimedia Appendix 12), with reduced heterogeneity (*I*^2^=69%; τ^2^=0.2895; *P*<.001) upon removal of outliers [24,28,45]. Even after removing studies with an overall high risk of bias and outliers, the effect size for physical function improvement remained small (*g*=–0.29; 95% CI –0.46 to –0.11; see “Sensitivity analysis omitted studies with high risk of bias and outliers of physical function” in Multimedia Appendix 12) and heterogeneity (*I*^2^=62%; τ^2^=0.0613; *P*<.001).

### Publication Bias

Visual analysis of funnel plots for physical activity did not reveal publication bias. However, asymmetry was noted in studies reporting pain relief and improvement in physical function (Multimedia Appendix 13 [24,26-32,45,47-54,56,57]). Notably, studies pertaining to pain relief and physical function tended to have smaller SEs corresponding with larger sample sizes. More included studies tended to have greater SMD. The study with a small sample size from Rafiq et al [28] might contribute to this asymmetry. However, quantitative tests were unable to identify publication bias for pain relief or physical function. Specifically, neither the Egger regression test nor the Begg rank correlation test found evidence of publication bias (Table 1). No studies were trimmed or imputed in analyzing pain relief and improvement in physical function.

## Discussion

### Key Results

We discovered low-certainty evidence suggesting that telehealth-supported programs for population with KOA might have a small benefit on pain, a minimal positive impact on physical activity, and a moderate benefit on physical function, even though the changes in these primary outcomes were not clinically meaningful. Furthermore, this review revealed moderate-certainty evidence that telehealth-supported exercise or physical activity programs could lead to a statistically significant improvement in quality of life and self-efficacy for pain, with low-certainty evidence that telehealth-supported interventions enhanced global improvement. Low-certainty evidence, however, demonstrated that telehealth-supported programs had little impact on improving self-efficacy for physical function. In studies involving interventions for patients and health care providers, better results were observed in the group using telehealth-supported exercise or physical activity programs. Moreover, greater effects of the telehealth-supported exercise or physical activity programs were observed when these programs were delivered via mobile apps, the internet, or a combination of both. These effects were particularly notable in studies where the control group received inactive interventions. These findings suggested a definite role of telehealth-supported exercise or physical activity programs in the management of KOA.

Multiple factors influenced the efficacy of telehealth-based interventions, leading to high heterogeneity. We performed influence and subgroup analysis to identify the source of heterogeneity, identifying Rafiq et al [28] as a significant contributor, where notable improvements were observed in outcome measurement in the intervention group. It is possible that extra improvements observed in the study resulted from clients being reminded to complete their exercise sessions [28]. Reminders, monitoring, and coaching are considered important components of telehealth-supported programs [63], our analysis found no significant differences in subgroups according to the presence of these elements. Counseling or coaching could be beneficial for health information communication and helping people to make decisions [64]. While in-person contact with clinicians is often viewed as essential for providing accessible expertise, it may be controversial to use virtual contact skills between remote clients and health care providers, such as consultations or coaching [65], targeted client alerts and reminders [66], and remote client monitoring [67]. To better understand the clinical significance of telehealth-supported exercise or physical activity programs and standardize those formats and components, further studies with rigorous designs should investigate the impact of remote consultations, coaching, reminders, and monitoring.

Our study identified that targeted primary users, digital technology, and comparators were more important than intervention components. Since the WHO classification (version 1.0) [33] is a useful and effective tool for identifying the particulars of telemedicine, we used it to categorize telehealth-based programs. Our findings underscore the importance of patient and health care provider communication in improving pain and physical function outcomes. Additionally, control interventions could potentially be a source of heterogeneity. Patients engaging in telehealth-supported exercise or physical activity programs demonstrated superior outcomes compared to inactive controls and achieved comparable outcomes in pain reduction and physical function improvement to active controls. Consequently, patients might be motivated by the significant advantages of telehealth results that are obtained at a reasonable cost and with ease, especially in circumstances where medical resources may be limited [68].

### Comparison With Other Studies

Several reviews have focused on digital health technologies in KOA, each offering unique insights. A narrative review [69] encompassing 91 studies found that digital health interventions were efficacious in enhancing patient education, promoting physical activity, and facilitating exercise interventions for patients with KOA. Similarly, another scoping review [70] concluded that digital health programs were comparably beneficial to traditional therapy in ameliorating pain, enhancing physical function, and improving quality of life outcomes. However, these reviews did not provide quantified outcomes.

Prior systematic reviews and meta-analyses have synthesized the treatment effect of telehealth-based exercise or physical activity programs. Xiang et al [20] analyzed that 6 RCTs incorporated 8 different telerehabilitation strategies. Yang et al [71] and Chen et al [22] included 9 studies on telehealth-based exercise interventions (as of June 2021) and 12 RCTs on technology-supported exercise programs (as of August 2020), respectively. In total, 11 RCTs about telehealth-supported programs were reviewed to analyze the effect on pain by McHugh et al [19]. In our previous work, which involved 4 studies, we reported a positive impact of internet-based rehabilitation programs on pain of patients with KOA but not on physical function [21]; the meta-analysis by Xiang et al [20] supported this conclusion. However, in this study, we found that both pain and physical function were positively affected by digital exercise or physical activity programs. The differences in conclusions between our previous and current meta-analyses may stem from the increased number of included studies and the diversity of telehealth-supported programs. Moreover, in contrast to the preceding 2 meta-analytical studies [22,71], our research yielded positive results concerning physical function and quality of life. The subgroup analysis on digital technology in this review aligned with the findings from Yang et al [71], suggesting that programs delivered via websites or telephones might yield superior outcomes. The review conducted by McHugh et al [19] specifically focused on the pain symptom and described the distinction between inactive and active comparators without incorporating quantitative analysis. In contrast, this study provided a comprehensive subgroup analysis and demonstrated that the intervention group exhibited superior outcomes in terms of pain and physical functions when compared to the inactive comparator.

In addition to pain and physical function, this study also explored how telehealth exercise or physical activity programs affect the physical activity level of patients with KOA, a topic not extensively discussed in the previous meta-analysis. By focusing specifically on digital exercise or physical activity programs for KOA and their therapeutic effect on physical activity performance, this study expanded upon prior knowledge by including updated evidence and providing a comprehensive summary regarding the effectiveness of telehealth-based exercise or physical activity programs. It was notable that telehealth-based exercise or physical activity interventions led to a slight but significant improvement in physical activity. Furthermore, we used the MIDs to evaluate the variation of pain, physical activity, and physical function rather than solely focusing on statistical significance. This approach holds greater significance in the context of clinical reasoning.

### Strengths and Limitations

This study has several key strengths. First, this review included telehealth-supported exercise or physical activity programs delivered through a wide variety of platforms, from SMS text messages and voice calls to internet-based applications or websites, providing a comprehensive understanding of the benefits of these programs. Second, the WHO classification of digital health interventions was introduced to label and describe the functions of each program, leading to new insight into subgroup analysis. Additionally, the study’s systematic review focused on the effect of telehealth-supported exercise or physical activity programs on physical activity and physical function with self-efficacy, resulting in new evidence and identifying crucial factors for future research, which could facilitate the development of more effective telehealth-supported exercise or physical activity programs. Nevertheless, this study has some limitations. First, included RCTs were heterogeneous due to the highly variable designs of telehealth programs. The high level of heterogeneity observed may have resulted from the difficulty in applying thorough blinding techniques in RCTs for telehealth. Nonetheless, by using multiple bias analysis, it was possible to partially identify the source of heterogeneity; consequently, the conclusion can be deemed strong and conclusive. Second, the long-term benefits of telehealth-supported exercise or physical activity programs remained unknown due to the limited number of studies that completed long-term assessments beyond 3 months.

### Future Research

Despite the established advantages of telehealth-supported exercise or physical activity programs, there are still obstacles to their widespread implementation as a first-line therapy for KOA, including the variation in technologies and management models. The WHO classification of digital health interventions aligns with the current intervention model and potential trend of digital technologies, which includes the nascent Internet of Things technologies, such as robotic-assisted training, wearable activity tracking, or visualized physical therapy [72]. Numerous recent experiments have used mobile apps and the internet, reflecting the growing trend toward using personal devices as telemedicine channels, in response to current interaction and technological advancements. Wearable devices, such as smartwatches or suits, are experiencing significant adoption, as they represent an innovative means of expanding health care services to everyday life situations. The rapid development of this technology, particularly direct-to-consumer wearable tracking technologies [73], holds promise for increasing patient acceptance and standardizing the application of remote technologies. There is a growing interest in various aspects related to remote technologies, such as the efficiency of transmitting information, the quality and consistency of interaction, and the cost or payment of new technologies [74]. These concerns may significantly facilitate the proliferation of telehealth-supported programs.

### Conclusions

Low-certainty evidence from this systematic review suggested that patients with KOA might benefit from telehealth-supported exercise or physical activity programs in terms of reduced pain intensity, increased physical activity, and improved physical function, although the improvement may not be clinically meaningful. Additionally, moderate-certainty evidence suggested that telehealth-supported programs targeting KOA led to improvements in quality of life and self-efficacy for pain. The general health condition of the population with KOA was improved by the telehealth-supported programs, though the level of certainty was low. However, this form of intervention was not effective in improving patients’ self-efficacy for physical function. Future research should consider the application of wearable technologies and expand the intervention duration to examine the long-term effect. Most significantly, further research should standardize norms of telehealth-supported exercise or physical activity programs to improve evidence for clinical practice.

## Appendix

Multimedia Appendix 1 PRISMA (Preferred Reporting Items for Systematic Reviews and Meta-aAnalyses) 2020 checklist.

Multimedia Appendix 2 Changes to the study protocol.

Multimedia Appendix 3 Search strategy.

Multimedia Appendix 4 List of studies excluded at full-text screening stage.

Multimedia Appendix 5 Characteristics of included studies.

Multimedia Appendix 6 Cochrane risk of bias graphical summary and Cochrane risk of bias summary per study.

Multimedia Appendix 7 PEDro scale quality assessment.

Multimedia Appendix 8 GRADE (Grading of Recommendations Assessment, Development, and Evaluation) summary of findings.

Multimedia Appendix 9 Forest plots of secondary outcomes.

Multimedia Appendix 10 Result of meta-regression test.

Multimedia Appendix 11 Results of outlier and influence analyses for primary outcomes.

Multimedia Appendix 12 Results of sensitivity analyses for primary outcomes.

Multimedia Appendix 13 Funnel plots of primary outcomes.

## Abbreviations

GRADE: Grading of Recommendations Assessment, Development, and Evaluation
KOA: knee osteoarthritis
KOOS: Knee Injury and Osteoarthritis Outcome Score
MID: minimally important difference
PICOS: Participants, Intervention, Comparison, Outcome, and Study Design
PRISMA: Preferred Reporting Items for Systematic Reviews and Meta-Analyses
RCT: randomized controlled trial
SMD: standardized mean difference
WHO: World Health Organization
WOMAC: Western Ontario and McMaster Universities Osteoarthritis Index

## References

[1] Hunter DJ, Bierma-Zeinstra S (2019). Osteoarthritis. Lancet.

[2] Kolasinski SL, Neogi T, Hochberg MC, Oatis C, Guyatt G, Block J, Callahan L, Copenhaver C, Dodge C, Felson D, Gellar K, Harvey WF, Hawker G, Herzig E, Kwoh CK, Nelson AE, Samuels J, Scanzello C, White D, Wise B, Altman RD, DiRenzo D, Fontanarosa J, Giradi G, Ishimori M, Misra D, Shah AA, Shmagel AK, Thoma LM, Turgunbaev M, Turner AS, Reston J (2020). 2019 American College of Rheumatology/Arthritis Foundation Guideline for the management of osteoarthritis of the hand, hip, and knee. Arthritis Care Res (Hoboken).

[3] Nguyen UDT, Zhang Y, Zhu Y, Niu J, Zhang B, Felson DT (2011). Increasing prevalence of knee pain and symptomatic knee osteoarthritis: survey and cohort data. Ann Intern Med.

[4] Vollset SE, Goren E, Yuan C, Cao J, Smith AE, Hsiao T, Bisignano C, Azhar GS, Castro E, Chalek J, Dolgert AJ, Frank T, Fukutaki K, Hay SI, Lozano R, Mokdad AH, Nandakumar V, Pierce M, Pletcher M, Robalik T, Steuben KM, Wunrow HY, Zlavog BS, Murray CJL (2020). Fertility, mortality, migration, and population scenarios for 195 countries and territories from 2017 to 2100: a forecasting analysis for the Global Burden of Disease Study. Lancet.

[5] Bijlsma JWJ, Berenbaum F, Lafeber FPJG (2011). Osteoarthritis: an update with relevance for clinical practice. Lancet.

[6] Caspersen CJ, Powell KE, Christenson GM (1985). Physical activity, exercise, and physical fitness: definitions and distinctions for health-related research. Public Health Rep.

[7] Kraus ViB, Sprow K, Powell KE, Buchner D, Bloodgood B, Piercy K, George SM, Kraus WE (2019). Effects of physical activity in knee and hip osteoarthritis: a systematic umbrella review. Med Sci Sports Exerc.

[8] Biver E, Berenbaum F, Valdes AM, Araujo de Carvalho I, Bindels LB, Brandi ML, Calder PC, Castronovo V, Cavalier E, Cherubini A, Cooper C, Dennison E, Franceschi C, Fuggle N, Laslop A, Miossec P, Thomas T, Tuzun S, Veronese N, Vlaskovska M, Reginster J, Rizzoli R (2019). Gut microbiota and osteoarthritis management: an expert consensus of the European Society for Clinical and Economic Aspects of Osteoporosis, Osteoarthritis and Musculoskeletal Diseases (ESCEO). Ageing Res Rev.

[9] Bannuru RR, Osani MC, Vaysbrot EE, Arden NK, Bennell K, Bierma-Zeinstra SMA, Kraus VB, Lohmander LS, Abbott JH, Bhandari M, Blanco FJ, Espinosa R, Haugen IK, Lin J, Mandl LA, Moilanen E, Nakamura N, Snyder-Mackler L, Trojian T, Underwood M, McAlindon TE (2019). OARSI guidelines for the non-surgical management of knee, hip, and polyarticular osteoarthritis. Osteoarthritis Cartilage.

[10] Beckwée D, Vaes P, Cnudde M, Swinnen E, Bautmans I (2013). Osteoarthritis of the knee: why does exercise work? a qualitative study of the literature. Ageing Res Rev.

[11] Skou ST, Pedersen BK, Abbott JH, Patterson B, Barton C (2018). Physical activity and exercise therapy benefit more than just symptoms and impairments in people with hip and knee osteoarthritis. J Orthop Sports Phys Ther.

[12] Allen KD, Golightly YM, White DK (2017). Gaps in appropriate use of treatment strategies in osteoarthritis. Best Pract Res Clin Rheumatol.

[13] Petursdottir U, Arnadottir SA, Halldorsdottir S (2010). Facilitators and barriers to exercising among people with osteoarthritis: a phenomenological study. Phys Ther.

[14] Nicolson PJA, Hinman RS, Kasza J, Bennell KL (2018). Trajectories of adherence to home-based exercise programs among people with knee osteoarthritis. Osteoarthritis Cartilage.

[15] Tuckson RV, Edmunds M, Hodgkins ML (2017). Telehealth. N Engl J Med.

[16] Catalyst NEJM (2018). What is telehealth?. NEJM Catalyst.

[17] Monaghesh E, Hajizadeh A (2020). The role of telehealth during COVID-19 outbreak: a systematic review based on current evidence. BMC Public Health.

[18] George MD, Danila MI, Watrous D, Reddy S, Alper J, Xie F, Nowell WB, Kallich J, Clinton C, Saag KG, Curtis JR (2021). Disruptions in rheumatology care and the rise of telehealth in response to the COVID-19 pandemic in a community practice-based network. Arthritis Care Res (Hoboken).

[19] McHugh C, Kostic A, Katz J, Losina E (2022). Effectiveness of remote exercise programs in reducing pain for patients with knee osteoarthritis: a systematic review of randomized trials. Osteoarthr Cartil Open.

[20] Xiang W, Wang J, Ji B, Li L, Xiang H (2023). Effectiveness of different telerehabilitation strategies on pain and physical function in patients with knee osteoarthritis: systematic review and meta-analysis. J Med Internet Res.

[21] Xie S, Wang Q, Wang L, Wang L, Song K, He C (2021). Effect of internet-based rehabilitation programs on improvement of pain and physical function in patients with knee osteoarthritis: systematic review and meta-analysis of randomized controlled trials. J Med Internet Res.

[22] Chen T, Or CK, Chen J (2021). Effects of technology-supported exercise programs on the knee pain, physical function, and quality of life of individuals with knee osteoarthritis and/or chronic knee pain: a systematic review and meta-analysis of randomized controlled trials. J Am Med Inform Assoc.

[23] Östlind E, Eek F, Stigmar K, Sant'Anna A, Hansson EE (2022). Promoting work ability with a wearable activity tracker in working age individuals with hip and/or knee osteoarthritis: a randomized controlled trial. BMC Musculoskelet Disord.

[24] Bennell KL, Lawford BJ, Keating C, Brown C, Kasza J, Mackenzie D, Metcalf B, Kimp AJ, Egerton T, Spiers L, Proietto J, Sumithran P, Harris A, Quicke JG, Hinman RS (2022). Comparing video-based, telehealth-delivered exercise and weight loss programs with online education on outcomes of knee osteoarthritis: a randomized trial. Ann Intern Med.

[25] Egerton T, Bennell KL, McManus F, Lamb KE, Hinman RS (2022). Comparative effect of two educational videos on self-efficacy and kinesiophobia in people with knee osteoarthritis: an online randomised controlled trial. Osteoarthritis Cartilage.

[26] Alasfour M, Almarwani M (2022). The effect of innovative smartphone application on adherence to a home-based exercise programs for female older adults with knee osteoarthritis in Saudi Arabia: a randomized controlled trial. Disabil Rehabil.

[27] Thiengwittayaporn S, Wattanapreechanon P, Sakon P, Peethong A, Ratisoontorn N, Charoenphandhu N, Charoensiriwath S (2023). Development of a mobile application to improve exercise accuracy and quality of life in knee osteoarthritis patients: a randomized controlled trial. Arch Orthop Trauma Surg.

[28] Rafiq MT, Abdul Hamid MS, Hafiz E (2021). The effect of rehabilitation protocol using mobile health in overweight and obese patients with knee osteoarthritis: a clinical trial. Adv Rheumatol.

[29] Nelligan RK, Hinman RS, Kasza J, Crofts SJC, Bennell KL (2021). Effects of a self-directed web-based strengthening exercise and physical activity program supported by automated text messages for people with knee osteoarthritis: a randomized clinical trial. JAMA Intern Med.

[30] Hsu YI, Chen YC, Lee CL, Chang NJ (2021). Effects of diet control and telemedicine-based resistance exercise intervention on patients with obesity and knee osteoarthritis: a randomized control trial. Int J Environ Res Public Health.

[31] Arfaei Chitkar SS, Mohaddes Hakkak HR, Saadati H, Hosseini SH, Jafari Y, Ganji R (2021). The effect of mobile-app-based instruction on the physical function of female patients with knee osteoarthritis: a parallel randomized controlled trial. BMC Womens Health.

[32] Allen KD, Woolson S, Hoenig HM, Bongiorni D, Byrd J, Caves K, Hall KS, Heiderscheit B, Hodges NJ, Huffman KM, Morey MC, Ramasunder S, Severson H, van Houtven C, Abbate LM, Coffman CJ (2021). Stepped exercise program for patients with knee osteoarthritis: a randomized controlled trial. Ann Intern Med.

[33] (2018). Classification of digital health interventions v1.0: a shared language to describe the uses of digital technology for health. World Health Organization.

[34] Page MJ, McKenzie JE, Bossuyt PM, Boutron I, Hoffmann TC, Mulrow CD, Shamseer L, Tetzlaff JM, Akl EA, Brennan SE, Chou R, Glanville J, Grimshaw JM, Hróbjartsson Asbjørn, Lalu MM, Li T, Loder EW, Mayo-Wilson E, McDonald S, McGuinness LA, Stewart LA, Thomas J, Tricco AC, Welch VA, Whiting P, Moher D (2021). The PRISMA 2020 statement: an updated guideline for reporting systematic reviews. BMJ.

[35] Andrade C (2020). Mean difference, standardized mean difference (SMD), and their use in meta-analysis: as simple as it gets. J Clin Psychiatry.

[36] Hoit G, Whelan DB, Dwyer T, Ajrawat P, Chahal J (2020). Physiotherapy as an initial treatment option for femoroacetabular impingement: a systematic review of the literature and meta-analysis of 5 randomized controlled trials. Am J Sports Med.

[37] Takeshima N, Sozu T, Tajika A, Ogawa Y, Hayasaka Y, Furukawa TA (2014). Which is more generalizable, powerful and interpretable in meta-analyses, mean difference or standardized mean difference?. BMC Med Res Methodol.

[38] Egger M, Davey Smith G, Schneider M, Minder C (1997). Bias in meta-analysis detected by a simple, graphical test. BMJ.

[39] Patsopoulos NA, Evangelou E, Ioannidis JP (2008). Sensitivity of between-study heterogeneity in meta-analysis: proposed metrics and empirical evaluation. Int J Epidemiol.

[40] Murad MH, Wang Z, Chu H, Lin L (2019). When continuous outcomes are measured using different scales: guide for meta-analysis and interpretation. BMJ.

[41] Devji T, Carrasco-Labra A, Qasim A, Phillips M, Johnston BC, Devasenapathy N, Zeraatkar D, Bhatt M, Jin X, Brignardello-Petersen R, Urquhart O, Foroutan F, Schandelmaier S, Pardo-Hernandez H, Vernooij RW, Huang H, Rizwan Y, Siemieniuk R, Lytvyn L, Patrick DL, Ebrahim S, Furukawa T, Nesrallah G, Schünemann HJ, Bhandari M, Thabane L, Guyatt GH (2020). Evaluating the credibility of anchor based estimates of minimal important differences for patient reported outcomes: instrument development and reliability study. BMJ.

[42] Sterne JAC, Savović J, Page MJ, Elbers RG, Blencowe NS, Boutron I, Cates CJ, Cheng H, Corbett MS, Eldridge SM, Emberson JR, Hernán MA, Hopewell S, Hróbjartsson A, Junqueira DR, Jüni P, Kirkham JJ, Lasserson T, Li T, McAleenan A, Reeves BC, Shepperd S, Shrier I, Stewart LA, Tilling K, White IR, Whiting PF, Higgins JPT (2019). RoB 2: a revised tool for assessing risk of bias in randomised trials. BMJ.

[43] Cashin AG, McAuley JH (2020). Clinimetrics: Physiotherapy Evidence Database (PEDro) Scale. J Physiother.

[44] Ebadi S, Henschke N, Forogh B, Nakhostin Ansari N, van Tulder MW, Babaei-Ghazani A, Fallah E (2020). Therapeutic ultrasound for chronic low back pain. Cochrane Database Syst Rev.

[45] Allen KD, Arbeeva L, Callahan LF, Golightly YM, Goode AP, Heiderscheit BC, Huffman KM, Severson HH, Schwartz TA (2018). Physical therapy vs internet-based exercise training for patients with knee osteoarthritis: results of a randomized controlled trial. Osteoarthritis Cartilage.

[46] Azma K, RezaSoltani Z, Rezaeimoghaddam F, Dadarkhah A, Mohsenolhosseini S (2018). Efficacy of tele-rehabilitation compared with office-based physical therapy in patients with knee osteoarthritis: a randomized clinical trial. J Telemed Telecare.

[47] Baker K, LaValley MP, Brown C, Felson DT, Ledingham A, Keysor JJ (2020). Efficacy of computer-based telephone counseling on long-term adherence to strength training in elderly patients with knee osteoarthritis: a randomized trial. Arthritis Care Res (Hoboken).

[48] Bennell K, Nelligan RK, Schwartz S, Kasza J, Kimp A, Crofts SJ, Hinman RS (2020). Behavior change text messages for home exercise adherence in knee osteoarthritis: randomized trial. J Med Internet Res.

[49] Bennell KL, Campbell PK, Egerton T, Metcalf B, Kasza J, Forbes A, Bills C, Gale J, Harris A, Kolt GS, Bunker SJ, Hunter DJ, Brand CA, Hinman RS (2017). Telephone coaching to enhance a home-based physical activity program for knee osteoarthritis: a randomized clinical trial. Arthritis Care Res (Hoboken).

[50] Bennell KL, Nelligan R, Dobson F, Rini C, Keefe F, Kasza J, French S, Bryant C, Dalwood A, Abbott JH, Hinman RS (2017). Effectiveness of an internet-delivered exercise and pain-coping skills training intervention for persons with chronic knee pain: a randomized trial. Ann Intern Med.

[51] Bennell KL, Schwartz S, Teo PL, Hawkins S, Mackenzie D, McManus F, Lamb KE, Kimp AJ, Metcalf B, Hunter DJ, Hinman RS (2022). Effectiveness of an unsupervised online yoga program on pain and function in people with knee osteoarthritis : a randomized clinical trial. Ann Intern Med.

[52] Gohir SA, Eek F, Kelly A, Abhishek A, Valdes AM (2021). Effectiveness of internet-based exercises aimed at treating knee osteoarthritis: the iBEAT-OA randomized clinical trial. JAMA Netw Open.

[53] Hinman RS, Campbell PK, Lawford BJ, Briggs AM, Gale J, Bills C, Kasza J, Harris A, French SD, Bunker SJ, Forbes A, Bennell KL (2020). Does telephone-delivered exercise advice and support by physiotherapists improve pain and/or function in people with knee osteoarthritis? telecare randomised controlled trial. Br J Sports Med.

[54] Li LC, Feehan LM, Xie H, Lu N, Shaw CD, Gromala D, Zhu S, Aviña-Zubieta JA, Hoens AM, Koehn C, Tam J, Therrien S, Townsend AF, Noonan G, Backman CL (2020). Effects of a 12-week multifaceted wearable-based program for people with knee osteoarthritis: randomized controlled trial. JMIR Mhealth Uhealth.

[55] Mecklenburg G, Smittenaar P, Erhart-Hledik JC, Perez DA, Hunter S (2018). Effects of a 12-week digital care program for chronic knee pain on pain, mobility, and surgery risk: randomized controlled trial. J Med Internet Res.

[56] O'Brien KM, Wiggers J, Williams A, Campbell E, Hodder RK, Wolfenden L, Yoong S, Robson E, Haskins R, Kamper S, Rissel C, Williams C (2018). Telephone-based weight loss support for patients with knee osteoarthritis: a pragmatic randomised controlled trial. Osteoarthritis Cartilage.

[57] Odole AC, Ojo OD (2013). A telephone-based physiotherapy intervention for patients with osteoarthritis of the knee. Int J Telerehabil.

[58] Odole AC, Ojo OD (2014). Is telephysiotherapy an option for improved quality of life in patients with osteoarthritis of the knee?. Int J Telemed Appl.

[59] Skrepnik N, Spitzer A, Altman R, Hoekstra J, Stewart J, Toselli R (2017). Assessing the impact of a novel smartphone application compared with standard follow-up on mobility of patients with knee osteoarthritis following treatment with hylan G-F 20: a randomized controlled trial. JMIR Mhealth Uhealth.

[60] Messier SP, Mihalko SL, Beavers DP, Nicklas BJ, DeVita P, Carr JJ, Hunter DJ, Lyles M, Guermazi A, Bennell KL, Loeser RF (2021). Effect of high-intensity strength training on knee pain and knee joint compressive forces among adults with knee osteoarthritis: the START randomized clinical trial. JAMA.

[61] Dupont J, Antonio L, Dedeyne L, O'Neill TW, Vanderschueren D, Rastrelli G, Maggi M, Bártfai G, Casanueva FF, Giwercman A, Słowikowska-Hilczer J, Punab M, Huhtaniemi IT, Wu FCW, Tournoy J, Koppo K, Gielen E (2021). Inflammatory markers are associated with quality of life, physical activity, and gait speed but not sarcopenia in aged men (40-79 years). J Cachexia Sarcopenia Muscle.

[62] Kim MS, Koh IJ, Choi KY, Sung YG, Park DC, Lee HJ, In Y (2021). The minimal clinically important difference (MCID) for the WOMAC and factors related to achievement of the MCID after medial opening wedge high tibial osteotomy for knee osteoarthritis. Am J Sports Med.

[63] Mosnaim GS, Stempel DA, Gonzalez C, Adams B, BenIsrael-Olive N, Gondalia R, Kaye L, Shalowitz M, Szefler S (2021). The impact of patient self-monitoring via electronic medication monitor and mobile app plus remote clinician feedback on adherence to inhaled corticosteroids: a randomized controlled trial. J Allergy Clin Immunol Pract.

[64] Gask L (2005). Role of specialists in common chronic diseases. BMJ.

[65] Wilhelm S, Weingarden H, Greenberg JL, Hoeppner SS, Snorrason I, Bernstein EE, McCoy TH, Harrison OT (2022). Efficacy of app-based cognitive behavioral therapy for body dysmorphic disorder with coach support: initial randomized controlled clinical trial. Psychother Psychosom.

[66] Mahmud N, Asch DA, Sung J, Reitz C, Coniglio MS, McDonald C, Bernard D, Mehta SJ (2021). Effect of text messaging on bowel preparation and appointment attendance for outpatient colonoscopy: a randomized clinical trial. JAMA Netw Open.

[67] Mehta SJ, Hume E, Troxel AB, Reitz C, Norton L, Lacko H, McDonald C, Freeman J, Marcus N, Volpp KG, Asch DA (2020). Effect of remote monitoring on discharge to home, return to activity, and rehospitalization after hip and knee arthroplasty: a randomized clinical trial. JAMA Netw Open.

[68] Duffy S, Lee TH (2018). In-person health care as option B. N Engl J Med.

[69] Shah N, Costello K, Mehta A, Kumar D (2022). Applications of digital health technologies in knee osteoarthritis: narrative review. JMIR Rehabil Assist Technol.

[70] Kitagawa T, Hayashi M (2023). mHealth for the self-management of knee osteoarthritis: scoping review. J Med Internet Res.

[71] Yang Y, Li S, Cai Y, Zhang Q, Ge P, Shang S, Han H (2023). Effectiveness of telehealth-based exercise interventions on pain, physical function and quality of life in patients with knee osteoarthritis: a meta-analysis. J Clin Nurs.

[72] Masuki S, Morikawa M, Nose H (2020). Internet of Things (IoT) system and field sensors for exercise intensity measurements. Compr Physiol.

[73] Fogel AL, Kvedar JC (2019). Reported cases of medical malpractice in direct-to-consumer telemedicine. JAMA.

[74] Romanick-Schmiedl S, Raghu G (2020). Telemedicine—maintaining quality during times of transition. Nat Rev Dis Primers.

