# Supplementary material for: Telehealth-Supported Exercise or Physical Activity Programs for Knee Osteoarthritis: Systematic Review and Meta-Analysis
Source: J Med Internet Res. 2024 Aug 2;26:e54876. doi: 10.2196/54876 (PMC11329855; doi:10.2196/54876)
Supplement: Multimedia Appendix 2 [file jmir_v26i1e54876_app2.docx]

1.1 Population in the included study changed into “participants regardless of age with a diagnosis of KOA”, rather than “participants aged 18 years or older, and diagnosed with knee osteoarthritis”.

1.2 A search update included the additional database of PubMed, and the specialist register GreyNet (http:// www.greynet.org/) and medRxiv (https://www.medrxiv.org/) for grey literature.

1.3 The search date updated into from inception to September 2023.
