# Supplementary material for: Telehealth-Supported Exercise or Physical Activity Programs for Knee Osteoarthritis: Systematic Review and Meta-Analysis
Source: J Med Internet Res. 2024 Aug 2;26:e54876. doi: 10.2196/54876 (PMC11329855; doi:10.2196/54876)
Supplement: Multimedia Appendix 3 [file jmir_v26i1e54876_app3.docx]

**Medline (Ovid) search strategy (1946 to August Week 4, 2023)**

|  | exp telerehabilitation/ |
| --- | --- |
|  | (Telecommunication or telemedicine or telehealth or telecoaching or E-health or E-medicine or Mobilehealth or Information technology or Information communication technology or Internet or Web-based or Videoconferencing or Remote consulting or Telemanagement or Teleconsulting or Telemonitoring or Mhealth or Online or SMS or Email or Wearable or Smartphone or Computer or Mobile or Remote). ab. |
|  | (Rehabilitation or Physical therapy or Exercise or physical activity).ab. |
|  | 1 or 2 or 3 |
|  | exp Osteoarthritis, Knee/ |
|  | (Knee osteoarthritis or Osteoarthritis of knee or Osteoarthritis of knee or KOA).ab. |
|  | 5 or 6 |
|  | Randomized controlled trial.pt. |
|  | Controlled clinical trial.pt. |
|  | (randomized or placebo or randomly or groups or trial).ab. |
|  | random*.ti,ab. |
|  | or/8-11 |
|  | 4 and 7 and 12 |

**EBM Reviews - Cochrane Central Register of Controlled Trials (Ovid) search strategy (to July 2023)**

|  | exp telerehabilitation/ |
| --- | --- |
|  | (Telecommunication or telemedicine or telehealth or telecoaching or E-health or E-medicine or Mobilehealth or Information technology or Information communication technology or Internet or Web-based or Videoconferencing or Remote consulting or Telemanagement or Teleconsulting or Telemonitoring or Mhealth or Online or SMS or Email or Wearable or Smartphone or Computer or Mobile or Remote). ab. |
|  | (Rehabilitation or Physical therapy or Exercise or physical activity).ab. |
|  | 1 or 2 or 3 |
|  | exp Osteoarthritis, Knee/ |
|  | (Knee osteoarthritis or Osteoarthritis of knee or Osteoarthritis of knee or KOA).ab. |
|  | 5 or 6 |
|  | Randomized controlled trial.pt. |
|  | Controlled clinical trial.pt. |
|  | (randomized or placebo or randomly or groups or trial).ab. |
|  | random*.ti,ab. |
|  | or/8-11 |
|  | 4 and 7 and 12 |

**Embase search strategy (1974 to 6^th^ September 2023)**

|  | exp telerehabilitation/ |
| --- | --- |
|  | (Telecommunication or telemedicine or telehealth or telecoaching or E-health or E-medicine or Mobilehealth or Information technology or Information communication technology or Internet or Web-based or Videoconferencing or Remote consulting or Telemanagement or Teleconsulting or Telemonitoring or Mhealth or Online or SMS or Email or Wearable or Smartphone or Computer or Mobile or Remote). ab. |
|  | (Rehabilitation or Physical therapy or Exercise or physical activity).ab. |
|  | 1 or 2 or 3 |
|  | exp Osteoarthritis, Knee/ |
|  | (Knee osteoarthritis or Osteoarthritis of knee or Osteoarthritis of knee or KOA).ab. |
|  | 5 or 6 |
|  | Randomized controlled trial.pt. |
|  | Controlled clinical trial.pt. |
|  | (randomized or placebo or randomly or groups or trial).ab. |
|  | random*.ti,ab. |
|  | or/8-11 |
|  | 4 and 7 and 12 |

**Scopus**

|  | TITLE-ABS-KEY ( telerehabilitation ) |
| --- | --- |
|  | TITLE-ABS-KEY ( telecommunication* OR telemedicine OR telehealth OR telecoaching OR e-health OR e-medicine OR mobilehealth OR information AND technology OR information AND communication AND technology OR internet OR web-based OR videoconferencing OR remote AND consulting OR telemanagement OR teleconsulting OR telemonitoring OR mhealth OR online OR sms OR email OR wearable OR smartphone OR computer OR mobile OR remote ) |
|  | Rehabilitation or Physical therapy or Exercise or physical activity |
|  | TITLE-ABS-KEY ( rehabilitation OR physical AND therapy OR exercise OR physical AND activity ) ) OR ( TITLE-ABS-KEY ( telecommunication* OR telemedicine OR telehealth OR telecoaching OR e-health OR e-medicine OR mobilehealth OR information AND technology OR information AND communication AND technology OR internet OR web-based OR videoconferencing OR remote AND consulting OR telemanagement OR teleconsulting OR telemonitoring OR mhealth OR online OR sms OR email OR wearable OR smartphone OR computer OR mobile OR remote ) ) OR ( TITLE-ABS-KEY ( telerehabilitation ) |
|  | TITLE-ABS-KEY ( knee AND osteoarthritis OR osteoarthritis AND of AND knee OR osteoarthritis AND of AND knee OR koa ) |
|  | TITLE-ABS-KEY ( knee AND osteoarthritis OR osteoarthritis AND of AND knee OR osteoarthritis AND of AND knee OR koa ) ) AND ( ( TITLE-ABS-KEY ( rehabilitation OR physical AND therapy OR exercise OR physical AND activity ) ) OR ( TITLE-ABS-KEY ( telecommunication* OR telemedicine OR telehealth OR telecoaching OR e-health OR e-medicine OR mobilehealth OR information AND technology OR information AND communication AND technology OR internet OR web-based OR videoconferencing OR remote AND consulting OR telemanagement OR teleconsulting OR telemonitoring OR mhealth OR online OR sms OR email OR wearable OR smartphone OR computer OR mobile OR remote ) ) OR ( TITLE-ABS-KEY ( telerehabilitation ) ) ) |

**PubMed**

|  | telerehabilitation[MeSH Terms] |
| --- | --- |
|  | telecommunications*[Title/Abstract] |
|  | telemedicine[Title/Abstract] |
|  | telehealth[Title/Abstract] |
|  | telehealthcare[Title/Abstract] |
|  | telecoaching[Title/Abstract] |
|  | e-health[Title/Abstract] |
|  | e-medicine[Title/Abstract] |
|  | mobilehealth[Title/Abstract] |
|  | information technology[Title/Abstract] |
|  | internet[Title/Abstract] |
|  | web-based[Title/Abstract] |
|  | videoconferencing[Title/Abstract] |
|  | remote consultation[Title/Abstract] |
|  | telemanagement[Title/Abstract] |
|  | teleconsultation[Title/Abstract] |
|  | telemonitoring[Title/Abstract] |
|  | mhealth[Title/Abstract] |
|  | online[Title/Abstract] |
|  | SMS[Title/Abstract] |
|  | Email[Title/Abstract] |
|  | Wearable[Title/Abstract] |
|  | Smartphone[Title/Abstract] |
|  | Computer[Title/Abstract] |
|  | Mobile[Title/Abstract] |
|  | Remote[Title/Abstract] |
|  | Rehabilitation[Title/Abstract] |
|  | Physical therapy[Title/Abstract] |
|  | Exercise[Title/Abstract] |
|  | 1 or 2 or 3 or 4 or 5 or 6 or 7 or 8 or 9 or 10 or 11 or 12 or 13 or 14 or 15 or 16 or 17 or 18 or 19 or 20 or 21 or 22 or 23 or 24 or 25 or 26 or 27 or 28 or 29 |
|  | Osteoarthritis, knee[Title/Abstract] |
|  | Knee Osteoarthritis[Title/Abstract] |
|  | Osteoarthritis of knee[Title/Abstract] |
|  | Osteoarthritis of the knee[Title/Abstract] |
|  | 31 or 32 or 33 or 34 |
|  | Meta-analysis[Publication Type] |
|  | Review[Publication Type] |
|  | Randomized controlled trail[Publication Type] |
|  | Meta analysis[MeSH Terms] |
|  | Meta-analysis[Title/Abstract] |
|  | Search*[Title/Abstract] |
|  | Randomized[Title/Abstract] |
|  | placebo[Title/Abstract] |
|  | 36 or 37 or 38 or 39 or 40 or 41 or 42 or 43 |
|  | 30 and 35 and 44 |

**Web of science**

|  | TS=clinical trial* |
| --- | --- |
|  | TS=controlled trial* |
|  | TS=random* |
|  | #1 or #2 or #3 |
|  | TS=Knee Osteoarthritis |
|  | TS=telerehabilitation |
|  | #4 AND #5 AND #6 |

**Physiotherapy Evidence Database (PEDro)**

<https://www.pedro.org.au/>: telerehabilitation and knee osteoarthritis.

**Additional records identified through other sources**

<http://www.opengrey.eu/search/request?q=greynet>: telerehabilitation and knee osteoarthritis.

<https://www.medrxiv.org/>: telerehabilitation and knee osteoarthritis.
