# Supplementary material for: Telehealth-Supported Exercise or Physical Activity Programs for Knee Osteoarthritis: Systematic Review and Meta-Analysis
Source: J Med Internet Res. 2024 Aug 2;26:e54876. doi: 10.2196/54876 (PMC11329855; doi:10.2196/54876)
Supplement: Multimedia Appendix 4 [file jmir_v26i1e54876_app4.docx]

| **Study title** | **Author and publication year** | **Reason for exclusion** |
| --- | --- | --- |
| Effectiveness of an Interactive, Multi-functional Mobile App-based Technology in Endogenous Healthcare for Individuals With or at Risk of Knee Osteoarthritis: A 12-month Randomized Controlled Trial | Kalun Or, et al. 2019 [1] | No accessible full text |
| MobilWise: Mobile Phone Remote Coaching After Worksite Joint ADventure Exposure | Not provided. 2016 [2] | No accessible full text |
| ZetrOZ Wearable Ultrasound Clinical Study | Ralph Ortiz, D.O et al. 2014 [3] | No accessible full text |
| SuPRA: Using Wearable Activity Trackers with a New Application to Improve Physical Activity in Knee Osteoarthritis | Navi Grewal et al. 2019 [4] | No accessible full text |
| Exercise therapy, manual therapy, or both, for management of osteoarthritis of the hip or knee: 2-year follow-up of a randomized clinical trial | Abbott, J. H, et al. 2014 [5] | No telerehabilitation |
| Feasibility and efficacy of remotely supervised cranial electrical stimulation for pain in older adults with knee osteoarthritis: A randomized controlled pilot study | Ahn, H, et al. 2020 [6] | Use neuromuscular electrical stimulation |
| Is a periodized circuit training delivered by telerehabilitation effective for patients with knee osteoarthritis? a phase I randomized controlled trial | Aily, J, et al. 2020 [7] | No effectiveness outcome |
| Efficacy of high intensity laser therapy in knee osteoarthritis: a double-blind controlled randomized study | Akaltun, M. S, et al. 2021 [8] | No telerehabilitation |
| Effects of Preoperative Telerehabilitation on Muscle Strength, Range of Motion, and Functional Outcomes in Candidates for Total Knee Arthroplasty: A Single-Blind Randomized Controlled Trial | An, J, et al. 2021 [9] | Patients received total knee arthroplasty |
| Treatment of knee osteoarthritis with platelet-rich plasma in comparison with transcutaneous electrical nerve stimulation plus exercise: a randomized clinical trial | Angoorani, H, et al. 2015 [10] | No telerehabilitation |
| App-based rehabilitation program after total knee arthroplasty: a randomized controlled trial | Bäcker, H. C, et al. 2021 [11] | Patients received total knee arthroplasty |
| The effect of exercise therapy on inflammatory activity assessed by MRI in knee osteoarthritis: Secondary outcomes from a randomized controlled trial | Bandak, E, et al. 2021 [12] | Not RCT |
| EFFECTS OF TECHNOLOGY-ENABLED PHYSICAL ACTIVITY COUNSELLING ON INTRA-INDIVIDUAL VARIABILITY AND OTHER MEASURES OF COGNITIVE PERFORMANCE AMONG ADULTS WITH KNEE OSTEOARTHRITIS | Best, J. R, et al. 2018 [13] | Psychological treatment |
| Effectiveness of a web-based physical activity intervention in patients with knee and/or hip osteoarthritis: randomized controlled trial | Bossen, D, et al. 2013 [14] | Patients with other osteoarthritis |
| Measuring and Improving Evidence-Based Patient Care Using a Web-Based Gamified Approach in Primary Care (QualityIQ): Randomized Controlled Trial | Burgon, T, et al. 2021 [15] | Patients with other diseases |
| The Effectiveness of Virtual Reality Rehabilitation in Patients with Knee and Hip Osteoarthritis | Byra, J.Czernicki, K, et al. 2020 [16] | Patients with other osteoarthritis |
| Effectiveness of Dry Needling Therapy on Pain, Hip Muscle Strength, and Physical Function in Patients With Hip Osteoarthritis: A Randomized Controlled Trial | Ceballos-Laita, L, et al. 2021 [17] | No telerehabilitation |
| Improving Maximal Strength in the Initial Postoperative Phase After Anterior Cruciate Ligament Reconstruction Surgery: Randomized Controlled Trial of an App-Based Serious Gaming Approach | Clausen, J. D, et al. 2020 [18] | Patients received anterior cruciate ligament reconstruction surgery |
| Analgesic knee orthosis applying transcutaneous nerve electrical stimulation operated by bluetooth system in knee osteoarthritis. | Cobo-Sevilla, V. 2018 [19] | Neuromuscular electrical stimulation |
| Reductions of cardiovascular and metabolic risk factors after a 14-week periodized training model in patients with knee osteoarthritis: a randomized controlled trial | de Almeida, A. C. 2021 [20] | No telerehabilitation |
| Impact of telephone reinforcement and negotiated contracts on behavioral predictors of exercise maintenance in older adults with osteoarthritis | Desai, P. M, et al. 2014 [21] | Patients with other osteoarthritis |
| Feasibility and preliminary effects of a tele-prehabilitation program and an in-person prehablitation program compared to usual care for total hip or knee arthroplasty candidates: a pilot randomized controlled trial | Doiron-Cadrin, P, et al. 2020 [22] | Patients received total Knee Arthroplasty |
| No effects of a 12-week supervised exercise therapy program on gait in patients with mild to moderate osteoarthritis: a secondary analysis of a randomized trial | Eitzen, I, et al. 2016 [23] | Not RCT |
| Rehabilitation program in osteoarthritis patients during the covid-19 lockdown | Fugaru, O, et al. 2021 [24] | Patients with other osteoarthritis |
| Effects of high-velocity resistance training on muscle function, muscle properties, and physical performance in individuals with hip osteoarthritis: a randomized controlled trial | Fukumoto, Y, et al. 2014 [25] | No telerehabilitation |
| The clinical effects of mobilization with passive ankle dorsiflexion using a passive ankle dorsiflexion apparatus on older patients with knee osteoarthritis: A randomized trial | Fung, K. W. Y, et al. 2021 [26] | No telerehabilitation |
| Lower limb muscle strengthening does not change frontal plane moments in women with knee osteoarthritis: A randomized controlled trial | Foroughi, N, et al. 2011 [27] | No telerehabilitation |
| Combined intra-articular corticosteroid and exercise in patients with knee osteoarthritis: A randomised trial | Henriksen, M. 2014 [28] | No telerehabilitation |
| Initiating range of motion exercises within 24 hours following total knee arthroplasty affects the reduction of postoperative pain: A randomized controlled trial | Iwakiri, K, et al. 2020 [29] | No telerehabilitation |
| Investigation of clinical effects of high- and low-resistance training for patients with knee osteoarthritis: a randomized controlled trial | Jan, M. H, et al. 2008 [30] | No telerehabilitation |
| Evaluation of a Novel e-Learning Program for Physiotherapists to Manage Knee Osteoarthritis via Telehealth: Qualitative Study Nested in the PEAK (Physiotherapy Exercise and Physical Activity for Knee Osteoarthritis) Randomized Controlled Trial | Jones, S. E, et al. 2021 [31] | Physiotherapists were randomized but patients were not |
| Application of heat and a home exercise program for pain and function levels in patients with knee osteoarthritis: A randomized controlled trial | Karadağ, S, et al. 2019 [32] | No telerehabilitation |
| Effects of Pilates training VS. Suspension training on quality of life in women with knee osteoarthritis: A randomized controlled trial | Karimi, N, et al. 2021 [33] | No telerehabilitation |
| A comparison of two manual physical therapy approaches and electrotherapy modalities for patients with knee osteoarthritis: A randomized three arm clinical trial | Kaya Mutlu, E, et al. 2018 [34] | No telerehabilitation |
| Effectiveness of a Blended Physical Therapist Intervention in People With Hip Osteoarthritis, Knee Osteoarthritis, or Both: A Cluster-Randomized Controlled Trial | Kloek, Corelien J. J, et al. 2018 [35] | Patients with other osteoarthritis |
| Association Between Therapeutic Alliance and Outcomes Following Telephone-Delivered Exercise by a Physical Therapist for People With Knee Osteoarthritis: Secondary Analyses From a Randomized Controlled Trial | Lawford, B. J, et al. 2021 [36] | Not RCT |
| "I Was Really Pleasantly Surprised": Firsthand Experience and Shifts in Physical Therapist Perceptions of Telephone-Delivered Exercise Therapy for Knee Osteoarthritis-A Qualitative Study | Lawford, B. J, et al. 2019 [37] | Not RCT |
| Kinesiotape and quadriceps strengthening with elastic band in women with knee osteoarthritis and overweight or obesity. A randomized clinical trial | León-Ballesteros, S. 2020 [38] | No telerehabilitation |
| Wearable transcutaneous electrical nerve stimulation (actiTENS®) is effective and safe for the treatment of knee osteoarthritis pain: a randomized controlled trial versus weak opioids | Maheu, E, et al. 2022 [39] | Use neuromuscular electrical stimulation |
| Effect of Neuromuscular Electrical Stimulation During Walking on Pain Sensitivity in Women With Obesity With Knee Pain: A Randomized Controlled Trial | Matsuse, H, et al. 2022 [40] | No telerehabilitation |
| Telephone interventions for co-morbid insomnia and osteoarthritis pain: The OsteoArthritis and Therapy for Sleep (OATS) randomized trial design | McCurry, S. M, et al. 2019 [41] | Psychological treatment |
| The efficacy of exergaming in patients with knee osteoarthritis: A randomized controlled clinical trial | Mete, E, et al. 2022 [42] | No telerehabilitation |
| Effects of a 12-Week Digital Care Program for Chronic Knee Pain on Pain, Mobility, and Surgery Risk: Randomized Controlled Trial | Mecklenburg, G, et al. 2018 [43] | Patients without not osteoarthritis |
| Exploring Attitudes and Experiences of People With Knee Osteoarthritis Toward a Self-Directed eHealth Intervention to Support Exercise: Qualitative Study | Nelligan, R. K, et al. 2020 [44] | Not RCT |
| Telerehabilitation is non-inferior to usual care following total hip replacement - a randomized controlled non-inferiority trial | Nelson, M, et al. 2020 [45] | Patients without osteoarthritis |
| Long term efficacy of mobilisation with movement on pain and functional status in patients with knee osteoarthritis: a randomised clinical trial | Nigam, A. 2021 [46] | No telerehabilitation |
| Internet Cognitive-Behavioral Therapy for Depression in Older Adults With Knee Osteoarthritis: A Randomized Controlled Trial | O'Moore K, A, et al. 2018 [47] | Psychological treatment |
| Comparative effects of proprioceptive and isometric exercises on pain intensity and difficulty in patients with knee osteoarthritis: A randomised control study | Ojoawo, A. O. et al. 2016 [48] | No telerehabilitation |
| Promoting work ability with a wearable activity tracker in working age individuals with hip and/or knee osteoarthritis: a randomized controlled trial | Östlind, Elin et al. 2022 [49] | Patients with other osteoarthritis |
| Effects of knee taping during functional activities in older people with knee osteoarthritis: A randomized controlled clinical trial | Park, K. N, et al. 2018 [50] | No telerehabilitation |
| Effect of the dr. Bart application on healthcare use and clinical outcomes in people with osteoarthritis of the knee and/or hip in the Netherlands; a randomized controlled trial | Pelle, T, et al. 2020 [51] | Patients with other osteoarthritis |
| Does tension applied in kinesio taping affect pain or function in older women with knee osteoarthritis? A randomised controlled trial | Pinheiro, Y. T, et al. 2020 [52] | No telerehabilitation |
| Level of participation in physical therapy or an internet-based exercise training program: associations with outcomes for patients with knee osteoarthritis | Pignato, M, et al. 2018 [53] | Not RCT |
| Effectiveness of an interactive virtual telerehabilitation system in patients after total knee arthoplasty: a randomized controlled trial | Piqueras, M, et al. 2013 [54] | Patients received total knee arthroplasty |
| Relationship Between Attitudes and Beliefs and Physical Activity in Older Adults With Knee Pain: Secondary Analysis of a Randomized Controlled Trial | Quicke, J. G, et al. 2017 [55] | No telerehabilitation |
| Dextrose prolotherapy for knee osteoarthritis: results of a randomized controlled trial | Rabago, D. P, et al. 2011 [56] | No telerehabilitation |
| Evaluation of the Combined Application of Neuromuscular Electrical Stimulation and Volitional Contractions on Thigh Muscle Strength, Knee Pain, and Physical Performance in Women at Risk for Knee Osteoarthritis: A Randomized Controlled Trial | Rabe, K. G, et al. 2018 [57] | No telerehabilitation |
| Automated Internet-based pain coping skills training to manage osteoarthritis pain: a randomized controlled trial | Rini, Christine et al. 2015 [58] | Patients with other osteoarthritis |
| Internet-based outpatient telerehabilitation for patients following total knee arthroplasty: a randomized controlled trial | Russell, T. G, et al. 2011 [59] | Patients received total knee arthroplasty |
| Use of a self-guided mindfulness mobile application to improve pain outcomes in individuals with knee osteoarthritis | Sylvester, J, et al. 2022 [60] | Psychological treatment |
| Adding web-based behavioural support to exercise referral schemes for inactive adults with chronic health conditions: the e-coachER RCT | Taylor, A. H, et al. 2020 [61] | Patients without osteoarthritis |
| A Smartwatch Paired With A Mobile Application Provides Postoperative Self-Directed Rehabilitation Without Compromising Total Knee Arthroplasty Outcomes: A Randomized Controlled Trial | Tripuraneni, K. R.et al. 2021 [62] | Patients received total knee arthroplasty |
| Decision aids can decrease decisional conflict in patients with hip or knee osteoarthritis: Randomized controlled trial | van Dijk, L. A, et al. 2021 [63] | No telerehabilitation |

**RCT: Randomized controlled trial**

**References in Multimedia Appendix 4**

1. Or, K., *Effectiveness of an Interactive, Multi-functional Mobile App-based Technology in Endogenous Healthcare for Individuals With or at Risk of Knee Osteoarthritis: A 12-month Randomized Controlled Trial*. 2019.

2. *MobilWise: Mobile Phone Remote Coaching After Worksite Joint ADventure Exposure*, U. Northwestern, Editor. 2016.

3. Ralph Ortiz, D.O., *ZetrOZ Wearable Ultrasound Clinical Study*, H. National Institutes of, H. National Institute on Minority, and D. Health, Editors. 2014.

4. Grewal, N., *Supporting Physical Activity and Reducing Sedentary Behavior in Arthritis*, U. Simon Fraser, et al., Editors. 2015.

5. Abbott, J.H., et al., *Exercise therapy, manual therapy, or both, for management of osteoarthritis of the hip or knee: 2-year follow-up of a randomized clinical trial.* Osteoarthritis and Cartilage, 2014. **22**: p. S51.

6. Ahn, H., et al., *Feasibility and efficacy of remotely supervised cranial electrical stimulation for pain in older adults with knee osteoarthritis: A randomized controlled pilot study.* Journal of Clinical Neuroscience, 2020. **77**: p. 128-133.

7. Aily, J., et al., *Is a periodized circuit training delivered by telerehabilitation effective for patients with knee osteoarthritis? a phase i randomized controlled trial.* Osteoarthritis and Cartilage, 2020. **28**: p. S468-S469.

8. Akaltun, M.S., et al., *Efficacy of high intensity laser therapy in knee osteoarthritis: a double-blind controlled randomized study.* Clin Rheumatol, 2021. **40**(5): p. 1989-1995.

9. An, J., et al., *Effects of Preoperative Telerehabilitation on Muscle Strength, Range of Motion, and Functional Outcomes in Candidates for Total Knee Arthroplasty: A Single-Blind Randomized Controlled Trial.* Int J Environ Res Public Health, 2021. **18**(11).

10. Angoorani, H., et al., *Treatment of knee osteoarthritis with platelet-rich plasma in comparison with transcutaneous electrical nerve stimulation plus exercise: a randomized clinical trial.* Med J Islam Repub Iran, 2015. **29**: p. 223.

11. Bäcker, H.C., et al., *App-based rehabilitation program after total knee arthroplasty: a randomized controlled trial.* Arch Orthop Trauma Surg, 2021. **141**(9): p. 1575-1582.

12. Bandak, E., et al., *The effect of exercise therapy on inflammatory activity assessed by MRI in knee osteoarthritis: Secondary outcomes from a randomized controlled trial.* The Knee, 2021. **28**: p. 256-265.

13. Best, J., et al., *EFFECTS OF TECHNOLOGY-ENABLED PHYSICAL ACTIVITY COUNSELLING ON INTRA-INDIVIDUAL VARIABILITY AND OTHER MEASURES OF COGNITIVE PERFORMANCE AMONG ADULTS WITH KNEE OSTEOARTHRITIS.* Alzheimer's & Dementia, 2018. **14**: p. P671-P672.

14. Bossen, D., et al., *Effectiveness of a web-based physical activity intervention in patients with knee and/or hip osteoarthritis: randomized controlled trial.* J Med Internet Res, 2013. **15**(11): p. e257.

15. Burgon, T., et al., *Measuring and Improving Evidence-Based Patient Care Using a Web-Based Gamified Approach in Primary Care (QualityIQ): Randomized Controlled Trial.* J Med Internet Res, 2021. **23**(12): p. e31042.

16. Byra, J. and K. Czernicki, *The Effectiveness of Virtual Reality Rehabilitation in Patients with Knee and Hip Osteoarthritis.* J Clin Med, 2020. **9**(8).

17. Ceballos-Laita, L., et al., *Effectiveness of Dry Needling Therapy on Pain, Hip Muscle Strength, and Physical Function in Patients With Hip Osteoarthritis: A Randomized Controlled Trial.* Arch Phys Med Rehabil, 2021. **102**(5): p. 959-966.

18. Clausen, J.D., et al., *Improving Maximal Strength in the Initial Postoperative Phase After Anterior Cruciate Ligament Reconstruction Surgery: Randomized Controlled Trial of an App-Based Serious Gaming Approach.* JMIR Serious Games, 2020. **8**(1): p. e14282.

19. Cobo-Sevilla, V., et al., *Analgesic knee orthosis applying transcutaneous nerve electrical stimulation operated by bluetooth system in knee osteoarthritis. [Spanish].* Investigacion Clinica (Venezuela), 2018. **59(Supplement 1)**: p. 192-197.

20. de Almeida, A.C., et al., *Reductions of cardiovascular and metabolic risk factors after a 14-week periodized training model in patients with knee osteoarthritis: a randomized controlled trial.* Clin Rheumatol, 2021. **40**(1): p. 303-314.

21. Desai, P.M., et al., *Impact of telephone reinforcement and negotiated contracts on behavioral predictors of exercise maintenance in older adults with osteoarthritis.* American Journal of Health Behavior. **38**(3): p. 465-77.

22. Doiron-Cadrin, P., et al., *Feasibility and preliminary effects of a tele-prehabilitation program and an in-person prehablitation program compared to usual care for total hip or knee arthroplasty candidates: a pilot randomized controlled trial.* Disabil Rehabil, 2020. **42**(7): p. 989-998.

23. Eitzen, I., et al., *No effects of a 12-week supervised exercise therapy program on gait in patients with mild to moderate osteoarthritis: a secondary analysis of a randomized trial.* Journal of Negative Results in BioMedicine, 2015. **14**(1): p. 5.

24. Fugaru, O., et al., *Rehabilitation program in osteoarthritis patients during the covid-19 lockdown.* Osteoporosis international, 2021. **Vol.32**(SUPPL 1): p. S128-S129p.

25. Fukumoto, Y., et al., *Effects of high-velocity resistance training on muscle function, muscle properties, and physical performance in individuals with hip osteoarthritis: a randomized controlled trial.* Clin Rehabil, 2014. **28**(1): p. 48-58.

26. Fung, K.W.Y., D.H.K. Chow, and W.C. Shae, *The clinical effects of mobilization with passive ankle dorsiflexion using a passive ankle dorsiflexion apparatus on older patients with knee osteoarthritis: A randomized trial.* J Back Musculoskelet Rehabil, 2021. **34**(6): p. 1007-1014.

27. Foroughi, N., et al., *Lower limb muscle strengthening does not change frontal plane moments in women with knee osteoarthritis: A randomized controlled trial.* Clinical Biomechanics, 2011. **26**(2): p. 167-174.

28. Henriksen, M., et al., *Combined intra-articular corticosteroid and exercise in patients with knee osteoarthritis: A randomised trial.* Arthritis and Rheumatology, 2014. **10)**: p. S639.

29. Iwakiri, K., et al., *Initiating range of motion exercises within 24 hours following total knee arthroplasty affects the reduction of postoperative pain: A randomized controlled trial.* Asia Pac J Sports Med Arthrosc Rehabil Technol, 2020. **21**: p. 11-16.

30. Jan, M.H., et al., *Investigation of clinical effects of high- and low-resistance training for patients with knee osteoarthritis: a randomized controlled trial.* Phys Ther, 2008. **88**(4): p. 427-36.

31. Jones, S.E., et al., *Evaluation of a Novel e-Learning Program for Physiotherapists to Manage Knee Osteoarthritis via Telehealth: Qualitative Study Nested in the PEAK (Physiotherapy Exercise and Physical Activity for Knee Osteoarthritis) Randomized Controlled Trial.* J Med Internet Res, 2021. **23**(4): p. e25872.

32. Karadağ, S., et al., *Application of heat and a home exercise program for pain and function levels in patients with knee osteoarthritis: A randomized controlled trial.* Int J Nurs Pract, 2019. **25**(5): p. e12772.

33. Karimi, N., K.J. Dehkordi, and R.M. Rizi, *Effects of Pilates training VS. Suspension training on quality of life in women with knee osteoarthritis: A randomized controlled trial.* J Bodyw Mov Ther, 2021. **27**: p. 737-745.

34. Kaya Mutlu, E., et al., *A comparison of two manual physical therapy approaches and electrotherapy modalities for patients with knee osteoarthritis: A randomized three arm clinical trial.* Physiother Theory Pract, 2018. **34**(8): p. 600-612.

35. Kloek, C.J.J., et al., *Effectiveness of a Blended Physical Therapist Intervention in People With Hip Osteoarthritis, Knee Osteoarthritis, or Both: A Cluster-Randomized Controlled Trial.* Phys Ther, 2018. **98**(7): p. 560-570.

36. Lawford, B.J., et al., *Association Between Therapeutic Alliance and Outcomes Following Telephone-Delivered Exercise by a Physical Therapist for People With Knee Osteoarthritis: Secondary Analyses From a Randomized Controlled Trial.* JMIR Rehabil Assist Technol, 2021. **8**(1): p. e23386.

37. Lawford, B.J., et al., *"I Was Really Pleasantly Surprised": Firsthand Experience and Shifts in Physical Therapist Perceptions of Telephone-Delivered Exercise Therapy for Knee Osteoarthritis-A Qualitative Study.* Arthritis Care Res (Hoboken), 2019. **71**(4): p. 545-557.

38. León-Ballesteros, S., et al., *Kinesiotape and quadriceps strengthening with elastic band in women with knee osteoarthritis and overweight or obesity. A randomized clinical trial.* Reumatol Clin (Engl Ed), 2020. **16**(1): p. 11-16.

39. Maheu, E., et al., *Wearable transcutaneous electrical nerve stimulation (actiTENS®) is effective and safe for the treatment of knee osteoarthritis pain: a randomized controlled trial versus weak opioids.* Ther Adv Musculoskelet Dis, 2022. **14**: p. 1759720x211066233.

40. Matsuse, H., et al., *Effect of Neuromuscular Electrical Stimulation During Walking on Pain Sensitivity in Women With Obesity With Knee Pain: A Randomized Controlled Trial.* Arch Phys Med Rehabil, 2022. **103**(9): p. 1707-1714.

41. McCurry, S.M., et al., *Telephone interventions for co-morbid insomnia and osteoarthritis pain: The OsteoArthritis and Therapy for Sleep (OATS) randomized trial design.* Contemp Clin Trials, 2019. **87**: p. 105851.

42. Mete, E. and Z. Sari, *The efficacy of exergaming in patients with knee osteoarthritis: A randomized controlled clinical trial.* Physiother Res Int, 2022. **27**(3): p. e1952.

43. Mecklenburg, G., et al., *Effects of a 12-Week Digital Care Program for Chronic Knee Pain on Pain, Mobility, and Surgery Risk: Randomized Controlled Trial.* J Med Internet Res, 2018. **20**(4): p. e156.

44. Nelligan, R.K., et al., *Exploring Attitudes and Experiences of People With Knee Osteoarthritis Toward a Self-Directed eHealth Intervention to Support Exercise: Qualitative Study.* JMIR Rehabil Assist Technol, 2020. **7**(2): p. e18860.

45. Nelson, M., et al., *Telerehabilitation is non-inferior to usual care following total hip replacement - a randomized controlled non-inferiority trial.* Physiotherapy, 2020. **107**: p. 19-27.

46. Nigam, A., K.H. Satpute, and T.M. Hall, *Long term efficacy of mobilisation with movement on pain and functional status in patients with knee osteoarthritis: a randomised clinical trial.* Clin Rehabil, 2021. **35**(1): p. 80-89.

47. O'Moore K, A., et al., *Internet Cognitive-Behavioral Therapy for Depression in Older Adults With Knee Osteoarthritis: A Randomized Controlled Trial.* Arthritis Care Res (Hoboken), 2018. **70**(1): p. 61-70.

48. Ojoawo, A.O., M.O. Olaogun, and M.A. Hassan, *Comparative effects of proprioceptive and isometric exercises on pain intensity and difficulty in patients with knee osteoarthritis: A randomised control study.* Technol Health Care, 2016. **24**(6): p. 853-863.

49. Östlind, E., et al., *Promoting work ability with a wearable activity tracker in working age individuals with hip and/or knee osteoarthritis: a randomized controlled trial.* BMC Musculoskelet Disord, 2022. **23**(1): p. 112.

50. Park, K.N. and S.H. Kim, *Effects of knee taping during functional activities in older people with knee osteoarthritis: A randomized controlled clinical trial.* Geriatr Gerontol Int, 2018. **18**(8): p. 1206-1210.

51. Pelle, T., et al., *Effect of the dr. Bart application on healthcare use and clinical outcomes in people with osteoarthritis of the knee and/or hip in the Netherlands; a randomized controlled trial.* Osteoarthritis Cartilage, 2020. **28**(4): p. 418-427.

52. Pinheiro, Y.T., et al., *Does tension applied in kinesio taping affect pain or function in older women with knee osteoarthritis? A randomised controlled trial.* BMJ Open, 2020. **10**(12): p. e041121.

53. Pignato, M., et al., *Level of participation in physical therapy or an internet-based exercise training program: associations with outcomes for patients with knee osteoarthritis.* BMC Musculoskelet Disord, 2018. **19**(1): p. 238.

54. Piqueras, M., et al., *Effectiveness of an interactive virtual telerehabilitation system in patients after total knee arthoplasty: a randomized controlled trial.* J Rehabil Med, 2013. **45**(4): p. 392-6.

55. Quicke, J.G., et al., *Relationship Between Attitudes and Beliefs and Physical Activity in Older Adults With Knee Pain: Secondary Analysis of a Randomized Controlled Trial.* Arthritis Care Res (Hoboken), 2017. **69**(8): p. 1192-1200.

56. Rabago, D., et al., *Dextrose prolotherapy for knee osteoarthritis: a randomized controlled trial.* Ann Fam Med, 2013. **11**(3): p. 229-37.

57. Rabe, K.G., et al., *Evaluation of the Combined Application of Neuromuscular Electrical Stimulation and Volitional Contractions on Thigh Muscle Strength, Knee Pain, and Physical Performance in Women at Risk for Knee Osteoarthritis: A Randomized Controlled Trial.* PM&R, 2018. **10**(12): p. 1301-1310.

58. Rini, C., et al., *Automated Internet-based pain coping skills training to manage osteoarthritis pain: a randomized controlled trial.* Pain, 2015. **156**(5): p. 837-848.

59. Russell, T.G., et al., *Internet-based outpatient telerehabilitation for patients following total knee arthroplasty: a randomized controlled trial.* J Bone Joint Surg Am, 2011. **93**(2): p. 113-20.

60. Sylvester, J., A. Knobloch, and M. Hess, *Use of a self-guided mindfulness mobile application to improve pain outcomes in individuals with knee osteoarthritis.* Clinical journal of sport medicine, 2022. **Vol.32**(2): p. 224p.

61. Taylor, A.H., et al., *Adding web-based behavioural support to exercise referral schemes for inactive adults with chronic health conditions: the e-coachER RCT.* Health Technol Assess, 2020. **24**(63): p. 1-106.

62. Tripuraneni, K.R., et al., *A Smartwatch Paired With A Mobile Application Provides Postoperative Self-Directed Rehabilitation Without Compromising Total Knee Arthroplasty Outcomes: A Randomized Controlled Trial.* J Arthroplasty, 2021. **36**(12): p. 3888-3893.

63. van Dijk, L.A., et al., *Decision aids can decrease decisional conflict in patients with hip or knee osteoarthritis: Randomized controlled trial.* World J Orthop, 2021. **12**(12): p. 1026-1035.
