# Supplementary material for: Telehealth-Supported Exercise or Physical Activity Programs for Knee Osteoarthritis: Systematic Review and Meta-Analysis
Source: J Med Internet Res. 2024 Aug 2;26:e54876. doi: 10.2196/54876 (PMC11329855; doi:10.2196/54876)
Supplement: Multimedia Appendix 5 [file jmir_v26i1e54876_app5.docx]

|  | Country | Sample size (subjects in telehealth) | Age (yrs), mean±SD, I vs. C | Female (%) | Intervention | Types of telehealth | Models of delivery | Intervention time (weeks) | Comparator | Time point | WHO Classification | Outcome | KOA severity  (NRS/VAS/K-L) |
| --- | --- | --- | --- | --- | --- | --- | --- | --- | --- | --- | --- | --- | --- |
| Bennell et al (2022) [24] | Australia | 415 (347) | 65.4±8.2 vs. 64.1±8.1 vs. 65.3±8.3 | 54.7 | Exercise, tele-education, PA, and self-management plus remote coaching and monitoring | Internet and electronics | Virtual contact | 24 | Tele-exercise and tele-education or diet and tele-education | Pre-intervention, 6th, 12th month follow-up | Targeted client communication  Personal health tracking  Telemedicine | NRS; WOMAC-function subscale; body weight; IPEQ; The AQoL-8D; DASS-21; Global rating of change overall; Satisfaction with care; Orthopedic surgeon appointment; Willingness to have surgery; Incidence of total knee joint replacement and knee arthroscopy surgery; Health economic data | NRS≥4 |
| Egerton et al (2022) [25] | Australia | 589 (296) | median (IQR):55 (50-62) vs. 54 (50-61) | 65.9 | How to increase PA | Internet | No interacting contact | 12 min | Education | Pre-intervention, Post-intervention | Targeted client communication | ASES -pain subscale;  BFMS for OA; The Credibility/Expectancy Questionnaire; Perceived importance of being physically active; Motivation to be physically active; Knee OA knowledge and beliefs; Hopefulness; Level of concern; Perceived need for surgery; Global overall change | ASES-pain score in baseline: 6.78±1.76 |
| Alasfour and Almarwani (2022) [26] | Saudi Arabia | 40 (20) | 53.7±4.0 vs. 55.2±4.6 | 100 | Isometric/isotonic quadriceps exercise plus remote monitoring | Mobile application | In-person | 6 | Same exercise plus paper materials | Pre-intervention, 3rd, 6th week follow-up | Targeted client communication  Telemedicine | Self-reported exercise Adherence; ANPRS; ArWOMAC-physical function; FTSST | APNRS≤7 |
| Thiengwittayaporn et al (2021) [27] | Thailand | 82 (40) | 62.2±6.8 vs. 63.0±9.7 | 89.0 | Exercise and education plus remote monitoring | Mobile application | No interacting contact | 4 | Education handout | Pre-intervention, post-intervention | Targeted client communication  Healthcare provider decision support  Telemedicine | Patient’s ability to correctly perform prescribed exercises; , ROM; KSS; KOOS; Sports and recreation activities | K-L grade≥2 |
| Rafiq et al (2021) [28] | England | 114 (76) | 53.5±4.4 vs. 54.0±4.4 vs. 52.9±4.6 | 56.1 | Exercise and education plus remote reminder or not | SMS | Virtual contact | 12 | Education | Pre-intervention, post-intervention | Targeted client communication | WOMAC-pain subscale; TUG; PSFS; Katz Index of Independence in ADL | K-L grade 2 or 3 |
| Nelligan et al (2021) [29] | Australia | 206 (103) | 60.3±8.2 vs. 59.0±8.5 | 61.2 | Exercises, and how to increase PA plus remote reminders | Internet and Mobile application | No interacting contact | 24 | Education | Pre-intervention, post-intervention | Targeted client communication | WOMAC-function subscale; NRS; KOOS; AQoL-6D; PASE; ASES; SEE; Global overall change; Satisfaction | NRS≥4 |
| Hsu et al (2021) [30] | China | 66 (22) | 65.6±3.9 vs. 64.2±4.1 vs. 66.0±3.9 | 60.6 | Exercise plus remote monitoring and coaching | Telephone and Internet | Virtual contact | 12 | Self-management | Pre-intervention, Post-intervention | Telemedicine | Bioelectrical impedance analysis; WOMAC; Blood biochemical analysis; TUG | K-L grade≤3  VAS≥4 |
| Arfaei (2021) [31] | Iran | 60 (31) | 57.8±8.6 vs. 58.5±6.3 | 100.0 | How to increase PA | Mobile application | No interacting contact  In-Person | 8 | Education via face to face | Pre-intervention, post-intervention | Targeted client communication | WOMAC; SF-36 | WOMAC-pain score in baseline:18.45±5.1 |
| Allen et al (2021) [32] | America | 345 (230) | 59.9±9.9 vs. 60.2±11.1 | 15.4 | Exercise, PA, and physical therapy plus coaching | Internet and Telephone | No interacting contact  Virtual contact  In-Person | 36 | Education via mail | Pre-intervention, 3rd, 6th, 9th month follow-up | Targeted client communication | WOMAC; 30-second chair stand test; 40-m fast-paced walk; TUG; Stair climbing test (12 steps); 6-minute walk test; PASE | NRS≥3 |
| Allen et al (2018) [45] | United States | 350 (142) | 65.3±11.5 vs. 65.7±10.3 vs. 64.3±12.2 | 71.7 | Self-directed exercise and reminders plus remote monitoring | Internet | No interacting contact | 48 | An appropriate home exercise program, physical therapy, and wait list | Pre-intervention, 4th, and 12th months follow-up | Targeted client communication  Personal health tracking | WOMAC; The 30-second chair stand; TUG; Two-minute step test; Unilateral stand time; Four-Stage Balance Test; PASE; Participants' Global Assessment of Change; Paticipaints' self-reported current minutes per week of stretching; Strengthening and aerobic exercise | WOMAC-pain score in baseline: 6.1±3.8 |
| Azma et al (2018) [46] | Iran | 54 (27) | 55±5.2 vs. 56±5.1 | 60.2 | Exercises, education, and hot pack plus remote reminder and coaching | Voice call | Virtual contact  In-Person | 6 | Hot pack, face-to-face exercises and education | Pre-intervention, 1st,6th month follow-up | Targeted client communication  Personal health tracking  Telemedicine | VAS; KOOS; WOMAC- function and pain subscale; ADL Symptom, Sport, QoL subscale | K-L grade＜4 |
| Baker et al (2020) [47] | Massachusetts | 104 (52) | 65.8±6.6  vs.  64.5±8.3 | 81.7 | Exercise, reminder plus remote coaching and monitoring | Telephone | Virtual contact  In-Person | 96 | Exercise and automated phone message reminder | Pre-intervention, post-intervention | Targeted client communication  Telemedicine | Exercise adherence; WOMAC-pain and function subscale; Functional performance assessments; Quadriceps and hamstring strength; Predetermined maximum constant velocity | WOMAC≥4 |
| Bennell et al (2020) [48] | Australia | 110 (56) | 61.7±6.7  vs.  62.9±6.8 | 67.3 | Self-directed exercise and reminders | SMSs | No interacting contact | 24 | Self-directed Exercise program advised by physical therapists | Pre-intervention, Post-intervention | Personal health tracking | EARS Section B; Number of days home exercises completed in the past week; Adherence to home exercise program three times per week; NRS; KOOS; AQoL-6D; ASES; BFMS; PASE; Physical Activity Scale for the Elderly; Participant-reported global overall change | NRS≥4 |
| Bennell et al (2017) [49] | Australia | 168 (84) | 61.1±6.9  vs.  63.4±7.8 | 63.1 | Exercise, education, and PA intervention by physical therapist plus telephone coaching and monitoring | Telephone | Virtual contact  In-Person | 24 | Exercise, education, and PA intervention by physical therapist | Pre-intervention, 6th, 12th, 18th month follow-up | Personal health tracking  Telemedicine | Self-report measures; WOMAC- function subscale; NRS pain on walking in past week; WOMAC-pain; AQoL-6D; PASE; AAS; Stepping duration and steps per day over 7 consecutive days; GROC | NRS≥4 |
| Bennell et al (2017) [50] | United States | 148 (74) | 60.8±6.5 vs. 61.5±7.6 | 56.1 | Exercise, education, and PA intervention plus telephone coaching and monitoring | Internet | Virtual contact | 36 | Education | Pre-intervention, post-intervention | Personal health tracking  Personal health tracking  Telemedicine | NRS; WOMAC-function and pain subscale; AQoL-6D; ASES; Pain Catastrophizing Scale; Coping Attempts Scale; Global change overall in pain, physical function | NRS≥4 |
| Bennell et al (2022) [51] | Australia | 212 (107) | 62.8±8.2 vs. 61.8±7.2 | 69.8 | Exercise and tele-education plus reminder | Internet | No interacting contact | 12 | Tele-education | Pre- intervention, 3rd, 6th month follow-up | Targeted client communication | NRS-pain during walking； NRS- over all knee pain； WOMAC-function, pain, stiffness subscale; DASS-21; AQoL-6D; global change in condition of knee; ASES-pain and other symptoms; BFMS; Activities-Specific Balance Confidence Scale score | NRS≥4 |
| Gohir et al (2021) [52] | England | 105 (48) | 65.2±9.7 vs. 68.0±8.6 | 67.1 | Exercise and education plus remote reminder and monitoring | Mobile application | Virtual contact | 6 | Usual care | Pre-intervention, post-intervention | Targeted client communication  Telemedicine | NRS; 30-second sit-to-stand test; TUG; WOMAC pain and function subscale; MSK-HQ; Maximum voluntary contraction of quadriceps and hamstring muscles; pressure pain threshold | K-L grade≥1 |
| Hinman et al (2020) [53] | Australia | 175 (87) | 62.4±9.1 vs. 62.5±8.1 | 62..9 | Exercise, education, and how to increase PA plus coaching or not | Telephone and Internet | Virtual contact | 24 | Education | Pre-intervention, 6th ,12th month follow-up | Targeted client communication  Telemedicine | NRS-overall pain, pain in walking; WOMAC function and pain subscale; ASES; BFMS for OA; PAES; Barriers to Physical Activity Scale; Benefits of Physical Activity Scale; AQoL-8D; Global changes in pain, physical function, and physical activity; Satisfaction; Cost-effectiveness; Adverse events and healthcare usage | NRS≥4 |
| Li et al (2020) [54] | Canada | 51 (26) | 65.0±8.0 vs. 64.8±9.0 | 82.4 | Education and how to PA plus remote monitoring coaching | Telephone, Internet, and electronics | Virtual contact  In-Person | 12 | Waiting list | Pre-intervention, 3.25th, 6.5th, and 9.75th month follow-up | Personal health tracking  Telemedicine | Mean daily MVPA time; Average daily time in purposeful activity; Average daily step count; KOOS; PIHS; Theory of Planned Behavior Questionnaire; PHQ-9; Self-Reported Habit Index | KOOS-pain score in baseline: 68.92±13.98 |
| O'Brien et al (2018) [56] | Australia | 119 (59) | 63.0±11.1 vs 60.2±13.9 | 62.2 | How to increase PA and self-management plus coaching | Telephone, and Internet | Virtual contact | 24 | Usual care | Pre-intervention, 2nd, 6th, 10th, 14th, 18th, 22nd, and 26th month follow-up | Targeted client communication  Telemedicine | MVPA; NRS; Weight; WOMAC; 12-item Shirt Form Health Survey, Pittsburgh Sleep Quality Index; Active Australia Survey; Diet; Alcohol Use Disorders Identification Test; smoking prevalence; Survey of Pain Attitudes; healthcare utilization; DASS-21; physical component of the Fear Avoidance Beliefs Questionnaire; Global Perceived Effect scale; Objective weight (kg) and waist circumference; BMI | NRS≥3 |
| Odole et al (2013) [57] | Nigeria | 50 (25) | 56.0±7.4 vs. 55.0 ± 7.8 | 48.0 | Exercise plus remote monitoring and coaching | Voice call | Virtual contact | 6 | Exercise | Pre-intervention, 0.5th, 1st, 1.5th month follow-up | Telemedicine | IKHOAM; VAS | VAS score in baseline: 55.26±17.94 (mm) |
| Odole et al (2014) [58] | Nigeria | 50 (25) | 56.0±7.4 vs. 55.0±7.8 | 48.0 | Exercise plus remote coaching | Mobile application and Voice call | Virtual contact | 6 | Exercise | Pre-intervention, 0.5th, 1st, 1.5th month follow-up | Targeted client communication  Telemedicine | WHOQoL-Bref | VAS score in baseline 55.26±17.94 (mm) |
| Skrepnik et al (2017) [59] | United States | 211 (107) | 61.6±9.5 vs. 63.6±9.3 | 50.2 | How to increase PA plus remote monitor | Mobile application and electronics | No interacting contact | 12 | How to increase PA | Pre-intervention, 0.25th, 1st, 3rd, 6th month follow-up | Targeted client communication  Personal health tracking | Steps per day; 6-minute walk test; Sleep-light, sound and duration; PAM-13; VAMS; TEAEs | NRS＜9 |
| Abbreviations: I = intervention group; C = control group; PA = physical activity; SMS = Short Message Service; app = application; ROM = range of motion; KSS = Knee Society scores; WOMAC = Western Ontario and McMaster Universities Osteoarthritis Index function subscale; NRS = numerical rating scale; TUG = Timed Up and Go text; MSK-HQ = the Arthritis Research UK Musculoskeletal Health Questionnaire; PSFS = Patient-Specific Functional Scale; SF-36 = Short form of health survey questionnaire; SQUASH = Short Questionnaire to Assess Health-enhancing physical activity; PAM-13 = Patient Activation Measure; MVPA = moderate-to-vigorous physical activity; PA: physical activity; PASE = Physical Activity Scale for the Elderly; AQoL = Assessment of Quality of Life instrument; PIHS = Partners in Health Scale; KOOS = Knee Injury and OA Outcome Score; PHQ-9 = Patient Health Questionnaire-9; BFMS = Brief Fear of Movement Scale; ANPRS = Arabic Numeric Pain Rating Scale; ArWOMAC = Arabic version of the reduced Western Ontario and McMaster universities osteoarthritis index; EARS = Exercise Adherence Rating Scale; VAS = visual analogue scale; ASES = Arthritis Self-efficacy Scale; VAMS = Visual Analog Mood Scale; TEAEs = Treatment-emergent adverse events; AAS = Active Australia Survey; WHOQoL-Bref = World Health Organisation Quality of Life-Bref; SEE = Self-Efficacy for Exercise scale; AKHOAM = Ibadan Knee/Hip Osteoarthritis Outcome Measure; GROC = global rating of change; DASS-21 = the 21-item Depression, Anxiety, and Stress Scale; IPEQ = Incidental and Planned Exercise Questionnaire; FTSST = five-times-sit-to-stand test; AQoL-6D = Assessment of quality of life-6 dimensions; AQoL-8D = Assessment of quality of life-8 dimensions | | | | | | | | | | | | | |
