# Supplementary figures and images for: Telehealth-Supported Exercise or Physical Activity Programs for Knee Osteoarthritis: Systematic Review and Meta-Analysis
Source: J Med Internet Res. 2024 Aug 2;26:e54876. doi: 10.2196/54876 (PMC11329855; doi:10.2196/54876)

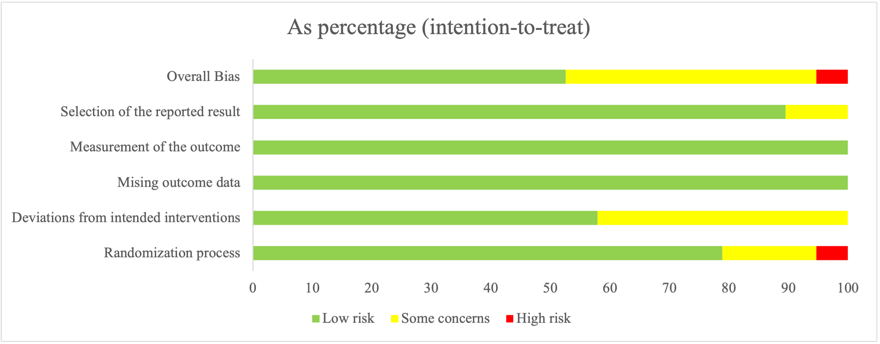


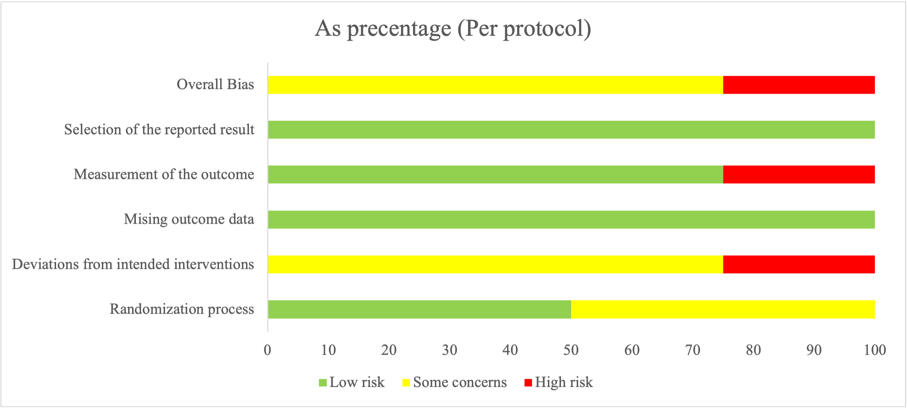


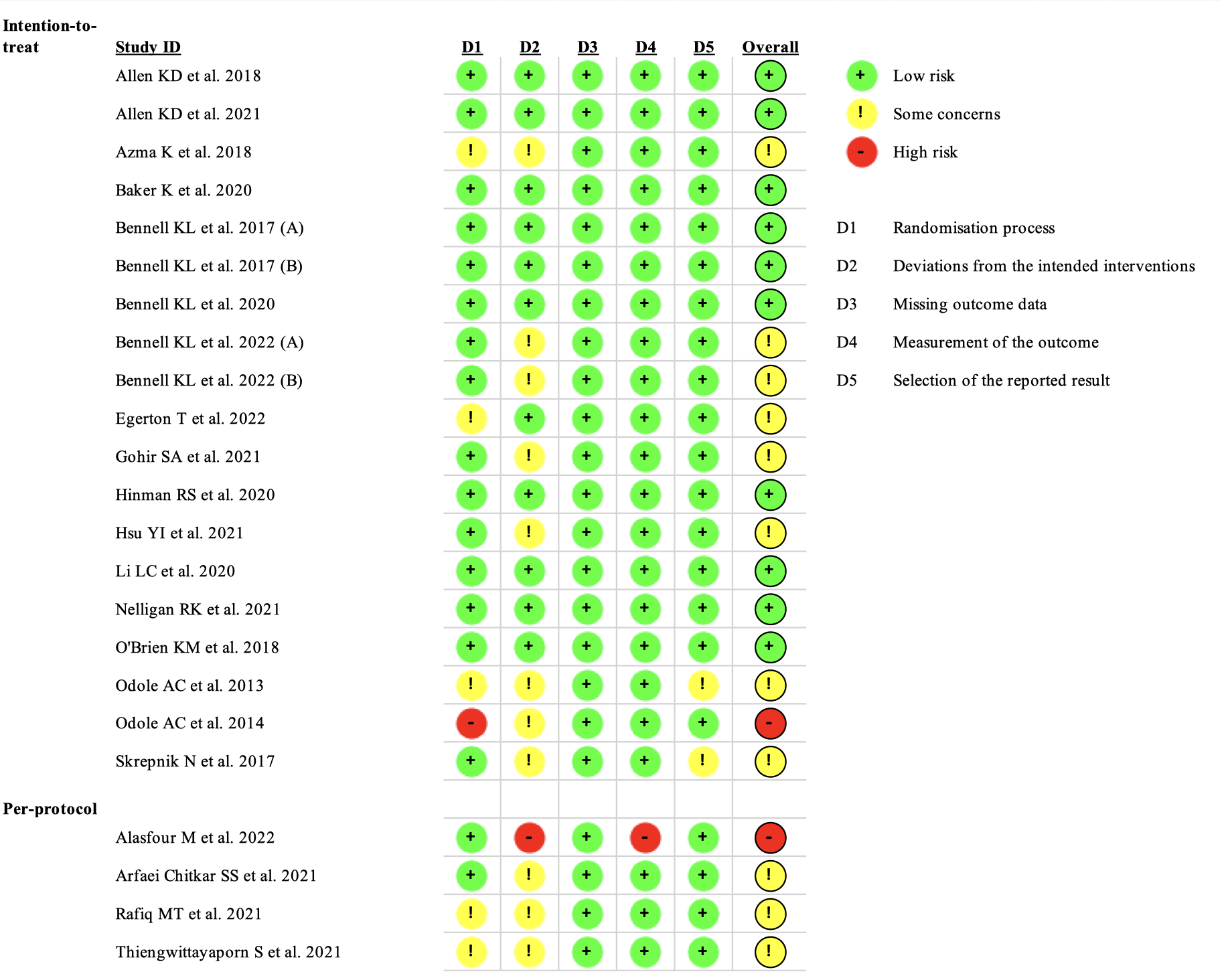

Supplement: Multimedia Appendix 6 [file jmir_v26i1e54876_app6.docx]

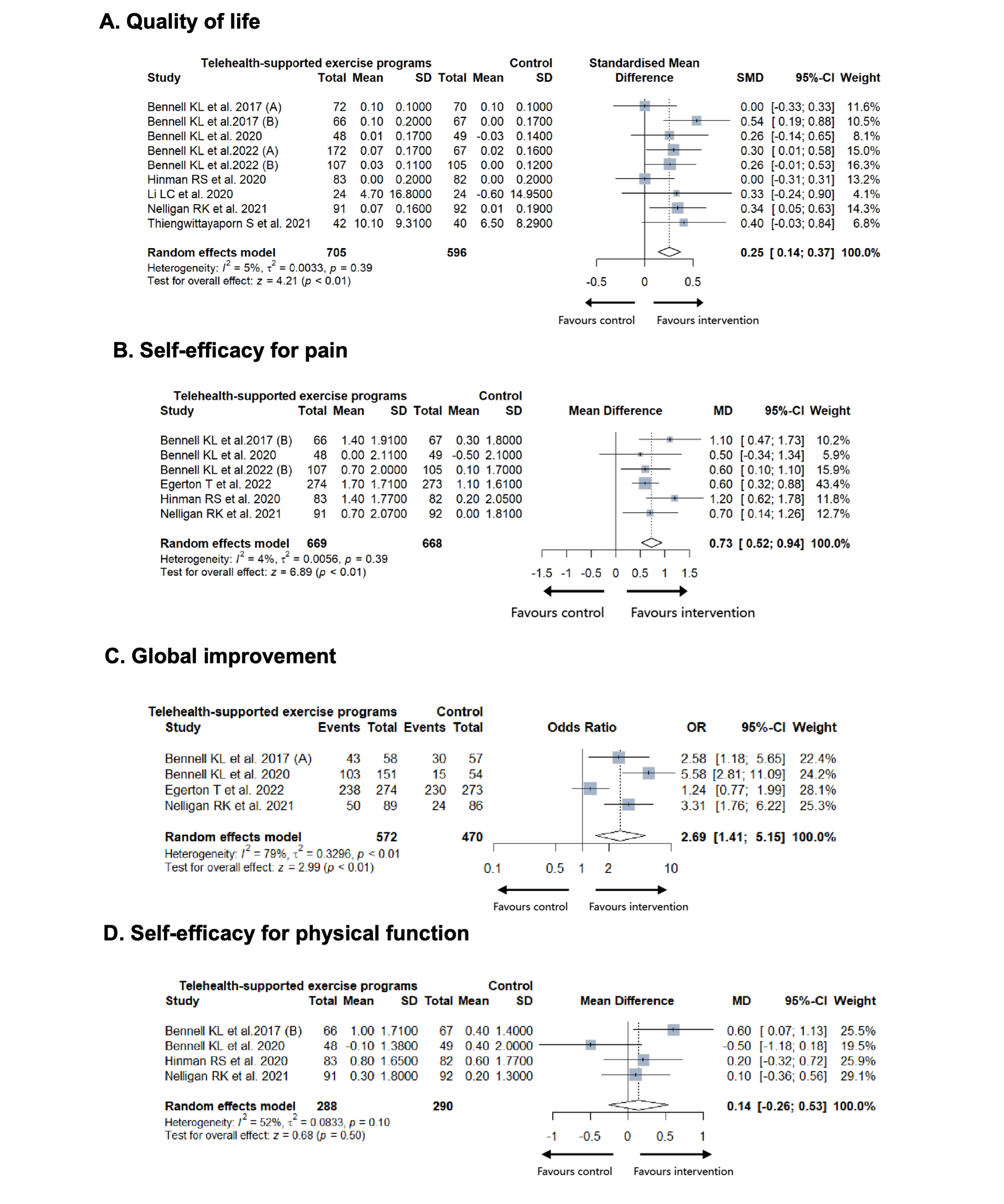

Supplement: Multimedia Appendix 9 [file jmir_v26i1e54876_app9.png]

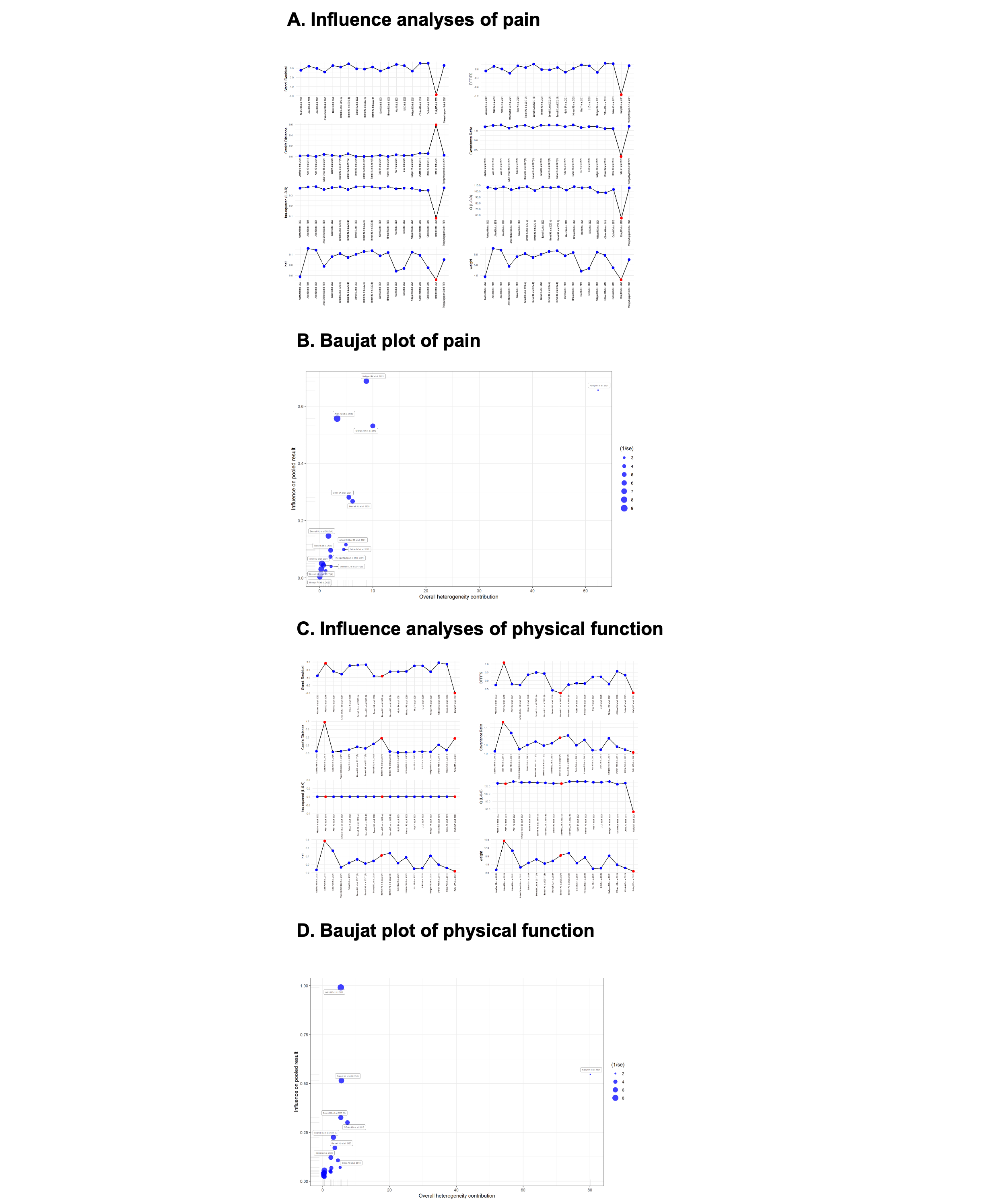

Supplement: Multimedia Appendix 11 [file jmir_v26i1e54876_app11.png]

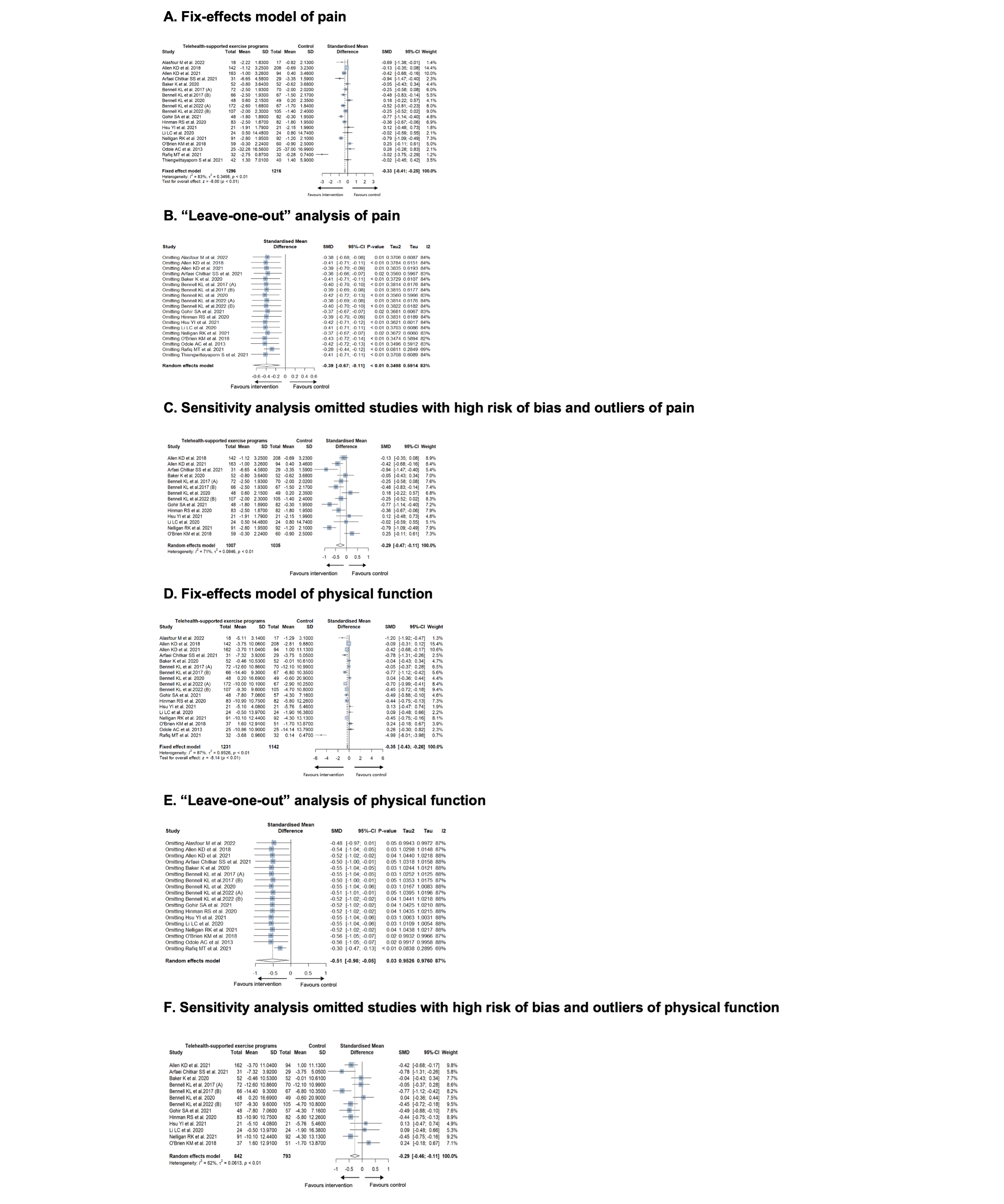

Supplement: Multimedia Appendix 12 [file jmir_v26i1e54876_app12.png]

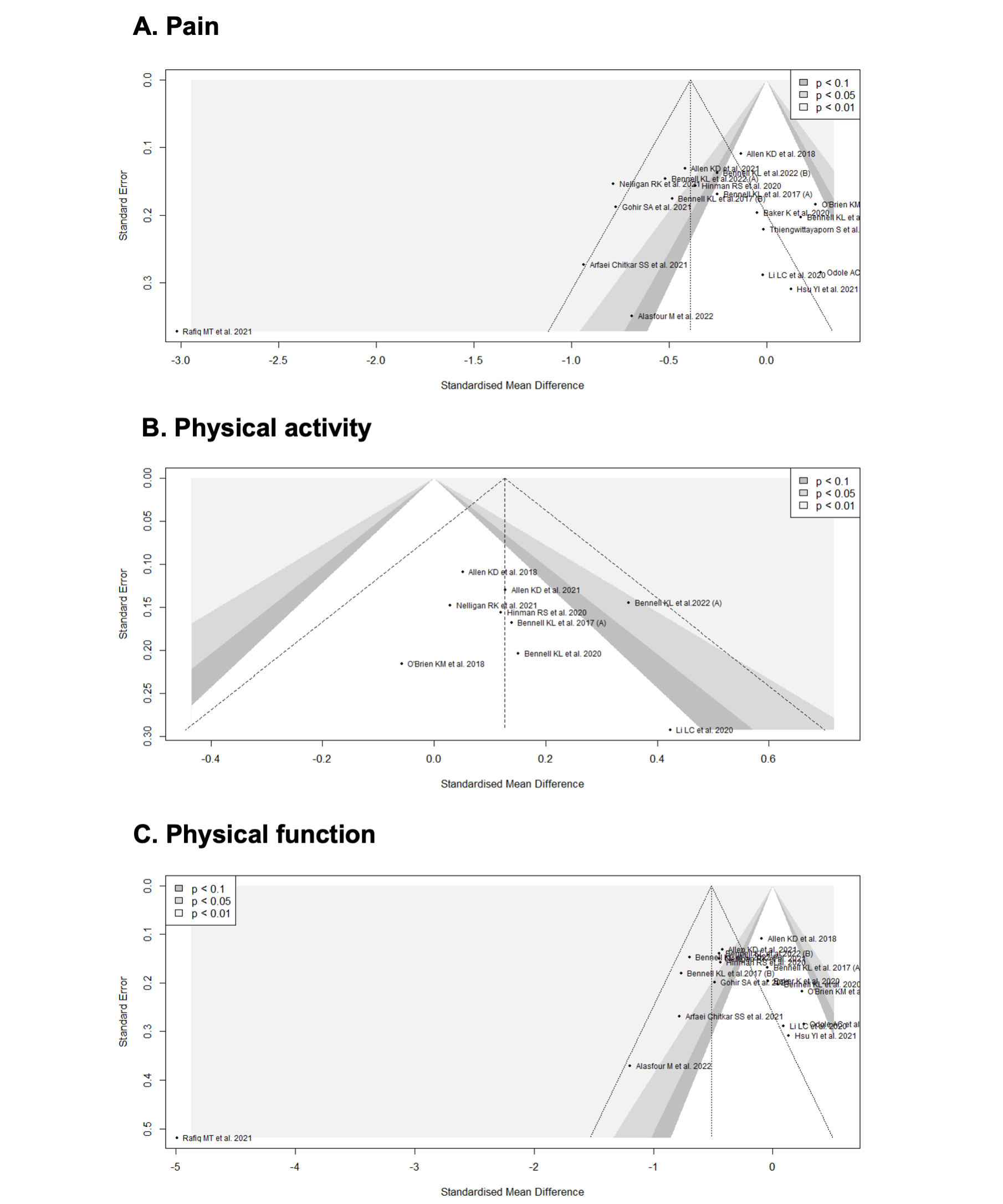

Supplement: Multimedia Appendix 13 [file jmir_v26i1e54876_app13.png]
