# Supplementary material for: Telehealth-Supported Exercise or Physical Activity Programs for Knee Osteoarthritis: Systematic Review and Meta-Analysis
Source: J Med Internet Res. 2024 Aug 2;26:e54876. doi: 10.2196/54876 (PMC11329855; doi:10.2196/54876)
Supplement: Multimedia Appendix 7 [file jmir_v26i1e54876_app7.docx]

|  | **Eligibility criteria specified (0/1)** | **Random allocation (0/1)** | **Concealed allocation (0/1)** | **Comparable at baseline (0/1)** | **Blinded subjects (0/1)** | **Blinded therapists (0/1)** | **Blinded assessors (0/1)** | **Adequate follow-up (0/1)** | **Intention-to-treat analysis (0/1)** | **Between group comparisons (0/1)** | **Point estimates and variability (0/1)** | **Summary** |
| --- | --- | --- | --- | --- | --- | --- | --- | --- | --- | --- | --- | --- |
| Alasfour and Almarwani (2022) [26] | 1 | 1 | 1 | 1 | 0 | 0 | 0 | 1 | 0 | 1 | 1 | 7 |
| Allen et al (2018) [45] | 1 | 1 | 1 | 1 | 0 | 0 | 1 | 1 | 1 | 1 | 1 | 9 |
| Allen et al (2021) [32] | 1 | 1 | 1 | 1 | 0 | 0 | 1 | 1 | 1 | 1 | 1 | 9 |
| Arfaei (2021) [31] | 1 | 0 | 1 | 1 | 0 | 0 | 1 | 1 | 1 | 1 | 1 | 8 |
| Azma et al (2018) [46] | 1 | 0 | 0 | 1 | 0 | 0 | 0 | 1 | 1 | 1 | 1 | 6 |
| Baker et al (2020) [47] | 1 | 1 | 1 | 1 | 0 | 0 | 1 | 1 | 1 | 1 | 1 | 9 |
| Bennell et al (2017, A) [49] | 1 | 1 | 1 | 1 | 1 | 0 | 1 | 1 | 1 | 1 | 1 | 10 |
| Bennell et al (2017, B) [50] | 1 | 1 | 1 | 1 | 0 | 0 | 1 | 1 | 1 | 1 | 1 | 9 |
| Bennell et al (2020) [48] | 1 | 1 | 1 | 1 | 1 | 0 | 1 | 1 | 1 | 1 | 1 | 10 |
| Bennell et al (2022, A) [24] | 1 | 1 | 1 | 1 | 0 | 0 | 0 | 1 | 1 | 1 | 1 | 8 |
| Bennell et al (2022, B) [51] | 1 | 1 | 1 | 1 | 0 | 0 | 0 | 1 | 1 | 1 | 1 | 8 |
| Egerton et al (2022) [25] | 1 | 1 | 1 | 1 | 1 | 0 | 1 | 1 | 0 | 1 | 1 | 9 |
| Gohir et al (2021) [52] | 1 | 1 | 1 | 1 | 0 | 0 | 0 | 1 | 1 | 1 | 1 | 8 |
| Hinman et al (2020) [53] | 1 | 1 | 1 | 1 | 0 | 0 | 1 | 1 | 1 | 1 | 1 | 9 |
| Hsu et al (2021) [30] | 1 | 0 | 1 | 1 | 1 | 0 | 0 | 1 | 1 | 1 | 1 | 8 |
| Li et al (2020) [54] | 1 | 1 | 1 | 1 | 0 | 0 | 1 | 1 | 1 | 1 | 1 | 9 |
| Nelligan et al (2021) [29] | 1 | 1 | 1 | 1 | 1 | 0 | 1 | 1 | 1 | 1 | 1 | 10 |
| O'Brien et al (2018) [56] | 1 | 0 | 1 | 1 | 1 | 0 | 1 | 1 | 1 | 1 | 1 | 9 |
| Odole et al (2013) [57] | 1 | 0 | 0 | 1 | 0 | 0 | 0 | 1 | 1 | 1 | 1 | 6 |
| Odole et al (2014) [58] | 1 | 1 | 0 | 1 | 0 | 0 | 0 | 1 | 1 | 1 | 1 | 7 |
| Rafiq et al (2021) [28] | 1 | 1 | 0 | 1 | 1 | 0 | 1 | 0 | 1 | 1 | 1 | 8 |
| Skrepnik et al (2017) [59] | 1 | 0 | 1 | 1 | 0 | 0 | 0 | 1 | 1 | 1 | 1 | 7 |
| Thiengwittayaporn et al (2021) [27] | 1 | 1 | 0 | 1 | 0 | 0 | 1 | 1 | 1 | 1 | 1 | 8 |
