# Supplementary material for: Telehealth-Supported Exercise or Physical Activity Programs for Knee Osteoarthritis: Systematic Review and Meta-Analysis
Source: J Med Internet Res. 2024 Aug 2;26:e54876. doi: 10.2196/54876 (PMC11329855; doi:10.2196/54876)
Supplement: Multimedia Appendix 8 [file jmir_v26i1e54876_app8.docx]

| **Telehealth-based exercise/physical activity programs for knee osteoarthritis** | | | | | | |
| --- | --- | --- | --- | --- | --- | --- |
| **Patient or population:** patients with knee osteoarthritis  **Settings:**  **Intervention:** Telerehabilitation | | | | | | |
| **Outcomes** | **Illustrative comparative risks* (95% CI)** | | **Relative effect**  **(95% CI)** | **No of Participants**  **(studies)** | **Quality of the evidence**  **(GRADE)** | **Comments** |
|  | Assumed risk | Corresponding risk |  |  |  |  |
|  | **Control** | **Telehealth-based exercise/physical activity programs** |  |  |  |  |
| **Pain** | The mean pain in the control group was between -37 and 1.4 from baseline | The mean pain in the intervention groups was **0.39 standard deviations lower** (0.67 to 0.11 lower) |  | 2512  (19 studies) | ⊕⊕⊝⊝ **low**^1,2^ | The methods measuring outcome of trials were different |
| **Physical activity** | The mean physical activity in the control group was between -21.9 and 84.8 from baseline | The mean physical activity in the intervention groups was **0.13 standard deviations higher** (0.03 to 0.23 higher) |  | 1570  (9 studies) | ⊕⊕⊝⊝ **low**^1,3^ | The methods measuring outcome of trials were different |
| **Physical function** | The mean physical function in the control group was between -13.14 and 1 from baseline | The mean physical function in the intervention groups was **0.51 standard deviations lower** (0.98 to 0.05 lower) |  | 2373 (18 studies) | ⊕⊕⊝⊝ **low**^1,4^ | The methods measuring outcome of trials were different |
| **Quality of life** | The mean pain in the control group was between -0.6 and 6.5 from baseline | The mean quality of life in the intervention groups was **0.25 standard deviations higher** (0.14 to 0.37 higher) |  | 1301 (9 studies) | ⊕⊕⊕⊝ **moderate**^1^ |  |
| **Self-efficacy for pain** | The mean pain in the control group was between -0.5 to 1.1 from baseline | The mean self-efficacy for pain in the intervention groups was **0.73 standard deviations higher** (0.52 to 0.94 higher) |  | 1337 (6 studies) | ⊕⊕⊕⊝ **moderate**^1^ |  |
| **Self-efficacy for physical function** | The mean pain in the control group was between 0.2 and 0.6 from baseline | The mean self-efficacy for physical function in the intervention groups was **0.14 standard deviations higher** (0.26 lower to 0.53 higher) |  | 578 (4 studies) | ⊕⊕⊝⊝ **low**^1,5^ |  |
| **Global improvement** | **636 per 1000** | **825 per 1000** (711 to 900) | OR 2.69 (1.41 to 5.15) | 1042 (4 studies) | ⊕⊕⊝⊝ low^1,6^ |  |
| *The basis for the **assumed risk** (e.g. the median control group risk across studies) is provided in footnotes. The **corresponding risk** (and its 95% confidence interval) is based on the assumed risk in the comparison group and the **relative effect** of the intervention (and its 95% CI).  **CI:** Confidence interval; **OR:** Odds ratio; | | | | | | |
| GRADE Working Group grades of evidence  **High quality:** Further research is very unlikely to change our confidence in the estimate of effect.  **Moderate quality:** Further research is likely to have an important impact on our confidence in the estimate of effect and may change the estimate.  **Low quality:** Further research is very likely to have an important impact on our confidence in the estimate of effect and is likely to change the estimate.  **Very low quality:** We are very uncertain about the estimate. | | | | | | |
| ^1^ Downgraded for risk of bias: Participants and personnel were unblinded  ^2^ Downgraded for inconsistency: Considerable heterogeneity (*I²*=83%)  ^3^ Downgraded for imprecision: Small sample size (Li LC et al. 2020)  ^4^ Downgraded for inconsistency: Considerable heterogeneity (*I²*=87%)  ^5^ Downgraded for inconsistency: Considerable heterogeneity (*I²*=52%)  ^6^ Downgraded for inconsistency: Considerable heterogeneity (*I²*=79%) | | | | | | |
