# Supplementary material for: Telehealth-Supported Exercise or Physical Activity Programs for Knee Osteoarthritis: Systematic Review and Meta-Analysis
Source: J Med Internet Res. 2024 Aug 2;26:e54876. doi: 10.2196/54876 (PMC11329855; doi:10.2196/54876)
Supplement: Multimedia Appendix 10 [file jmir_v26i1e54876_app10.docx]

| Relative factor | P-value of factor and pain | P-value of factor and physical function |
| --- | --- | --- |
| The existing of coaching | 0.1196 | 0.156 |
| The existing of monitoring | 0.2806 | 0.3102 |
| The existing of reminder | 0.3208 | 0.4046 |
| The content in experimental group | 0.9799 | 0.7398 |
| The model of delivery | 0.589 | 0.0726 |
| The type of tele-technology | **0.003** | **0.0115** |
| Intervention duration | 0.5575 | 0.0665 |
| The WHO classification | **0.0392** | **0.0399** |
| Active/inactive control | **0.0214** | 0.0663 |
| Sample size | 0.3215 | 0.4410 |
| Quality of study | 0.4922 | 0.6948 |
| Region | 0.2706 | 0.2060 |

WHO: World Health Organization
